# Distribution, inducibility, and characterisation of prophages in *Latilactobacillus sakei*

**DOI:** 10.1186/s12866-022-02675-y

**Published:** 2022-11-08

**Authors:** Conrad L. Ambros, Matthias A. Ehrmann

**Affiliations:** Lehrstuhl für Technische Mikrobiologie, Technische Universität München (TUM), 85354 Freising, Germany

**Keywords:** *Latilactobacillus sakei*, Prophages, Induction

## Abstract

**Background:**

Lactic acid bacteria (LAB) are used as starters in a wide variety of food fermentations. While the number of reports of phages infecting other LAB steadily increased over the years, information about phage associated with *Latilactobacillus sakei*, a frequently used meat starter, remains scarce.

**Results:**

In this study, a predictive genomic analysis of 43 *Latilactobacillus sakei* genomes revealed the presence of 26 intact, eleven questionable and 52 incomplete prophage sequences across all analysed genomes with a range of one to five predicted prophage sequences per strain. Screening 24 *sakei* strains for inducible prophages by utilising UV light or mitomycin C, we identified seven lysogenic strains showing lysis after induction during subsequent growth monitoring.

Electron microscopic analysis revealed fully assembled virions in the purified lysates of four samples, thus confirming successful prophage induction. All virions featured icosahedral, isomeric heads and long, most likely non-contractile tails indicating siphoviruses. By performing phylogenetic analyses with various marker genes as well as full prophage sequences, we displayed a remarkably high diversity of prophages, that share a similar gene module organisation and six different chromosomal integration sites were identified. By sequencing viral DNA purified from lysates of *Latilactobacillus sakei* TMW 1.46, we demonstrate that simultaneous induction of multiple prophages is possible.

**Conclusions:**

With this work, we not only provide data about the incidence of prophages harboured by the meat starter *Latilactobacillus sakei,* we also demonstrated their potential to impact growth of their host after induction, as well as forming seemingly fully assembled virions.

**Supplementary Information:**

The online version contains supplementary material available at 10.1186/s12866-022-02675-y.

## Background

After being patented by Jensen et al. in 1940 [1], the idea of adding pure cultures or mixtures of lactobacilli to comminuted raw meat, with the object of, amongst others, controlling and improving the organoleptic properties of raw sausage, led to experimentations utilising different species and strain combinations within the last 80 years.

Nowadays, lactic acid bacteria (LAB) are common representatives in sausage fermentation, next to Coagulase-negative staphylococci (CNS), yeasts and moulds [2]. Hereof, *Latilactobacillus sakei* is routinely used in meat fermentation, where some strains show high potential to control pathogens like *Listeria monocytogenes* and *Staphylococcus aureus* due to bacteriocin-production while providing important precursors for the formation of volatile and sensorial compounds as a result of its hydrolytic activity [3]. In the food industry production on large scale and semi-sterile conditions enable phage attacks, leading to lower product quality and thus economic losses as non-satisfactory products have to be discarded [4].

Only scarce information about phage connected to *Latilactobacillus sakei* and their impact on production of fermented meat products has been published so far. A previous study reported the influence of the virulent Lactiplantibacillus plantarum phage B_2_ in meat fermentation [5]. Authors discovered immediate growth suppression of the LAB starter culture and a delayed lactic acid production during the early fermentation days. While they showed that some of the surviving starter culture cells were still able to grow and ferment the sausage belatedly, they also gave their concerns about the negative impact of phages in the fermentation process when other *Lactobacillus* strains were used as starter cultures. They conclude that due to the observed delays in the fermentation process and tight schedules of producers of fermented meat products, phages might cause economically harm. To date, phage PWH2, isolated from fermented sausage, remains the only described phage connected to *L. sakei* [6]. PWH2 was discussed to be temperate as it granted surviving isolates of the previously infected host strain superinfection immunity against PWH2. Furthermore, one of the survivors showed inducible lysis after induction with mitomycin C. Unfortunately, the described phage got lost over the years and no genomic sequence data is available.

Fortunately, *Latilactobacillus sakei* was included in a recent publication where authors scanned for and described prophages in 16 different *Lactobacillus* species, predicting also intact prophages in *sakei* [7].

In a previous study we identified a prophage in the genome of *L. sakei* TMW 1.1398 [8]. Although classified as intact by PHASTER and UV-inducible partial lysis of the host cell, no fully assembled virulent phage particles were produced due to a transposase integration in the phage tail protein.

Phages replicate via the lytic cycle (virulent phages) leading to host cell lysis and release of phage progeny or replicate using both lytic and lysogenic cycles (temperate phages). Temperate phages are able to integrate into the bacterial genome (lysogeny). This state form of a bacteriophage in lysogeny is called a prophage. It replicates with its host cell while neither lysing the host cell nor generating phage particles. But, lysogeny can be reversed by external influences (induction) and the prophage genome induces lysis of the infected host, producing new phages. Primarily complete prophage genomes are subject to evolutionary degeneration and therefore often fragmentary and not or only partially functional (cryptic prophage). Thus, lysis of starter cultures can be caused by a phage attack by a virulent phage coming from the environment or by the induction of prophages or their remains [9].

Moreover, it is assumed that the host cell selects phage-associated functions, insofar as these gives an advantage to the host. These remaining sequences, referred to as “domesticated prophages”, often encode functions such as gene transfer and lysis by cell wall lytic enzymes [10].

It can be assumed that the presence of (cryptic) prophages under certain conditions has adverse consequences for the host and consequently also for the course of a fermentation. In addition, however, they probably also have an influence on the competitiveness within a microbial community. The aim of this study is to analyse the frequency as well as the degree of intactness of prophages and their inducibility in genomes of *L. sakei*.

## Results

### Prophage prediction in *Latilactobacillus sakei*

The majority of *L. sakei* strains used in this study during the screening are starter cultures in meat fermentation or were isolated from meat/sausage (Table 1), albeit strain TMW 1.411 was isolated from sauerkraut and strain TMW 1.1239 from a wheat sourdough fermentation. For the identification of prophage genomes in *L. sakei* with PHASTER [11, 12], we included all *sakei* genomes with the assembly levels “chromosome” and “complete” uploaded at the NCBI website so far, including mostly genomes of strains isolated from meat, meat products and fermented vegetables (e.g. kimchi). In total, 47 *sakei* genomes were considered, albeit four of the genomes were excluded due to having high nucleotide similarities to one another. This comprised the genome of strain TMW 1.578, showing a high nucleotide similarity to the genome of strain TMW 1.114 (100.00% ANIb / 99.75% of aligned nucleotides), as well as the genomes of the strains ZFM220, ZFM225 and ZFM229, being practically identical to the genome of strain LZ217 (100.00% ANIb / 99.72 - 99.77% of aligned nucleotides). Hence, 43 remaining genomes were analysed via PHASTER.

**Table 1:** *Latilactobacillus sakei* strains used in this study, the number of prophage sequences in strains, where genome data was available (for accession numbers see chapter “Availability of data and materials”), including completeness evaluation of those prophages (i, intact; q, questionable; inc, incomplete) as predicted by PHASTER and whether the strain showed lysis after induction via UV light or mitomycin C (+, strong lysis; (+), weak lysis; −, no visible lysis)

*Latilactobacillus sakei* strain	Isolation source (reference / origin)	Number of detected prophages			Inducibility	
		i	q	inc	UV light	Mitomycin C
23 K	Meat (Berthier et al. 1996)	0	0	2	–	–
C21B	Artisanal dry-fermented salami (Italy)	1	0	1	nd^a^	nd
C22G	Artisanal dry-fermented salami (Italy)	0	0	1	nd	nd
CBA3614	Kimchi (South Korea)	0	0	2	nd	nd
CBA3635	Fermented vegetable (South Korea)	2	0	1	nd	nd
DS4	Korean Kimchi (South Korea)	1	0	0	nd	nd
DSM 20017 T	Moto (Katagiri et al. 1934; Torriani et al. 1996)	0	1	2	–	–
E23B	Artisanal dry-fermented salami (Italy)	0	0	1	nd	nd
E28G	Artisanal dry-fermented salami (Italy)	1	0	1	nd	nd
FAM18311	Food (Switzerland)	1	0	0	nd	nd
FLEC01	Human feces	0	0	1	nd	nd
J112	French dry-type pork sausage	0	1	1	nd	nd
J156	French dry-type pork sausage	0	0	2	nd	nd
J160x1	Horse meat	0	0	1	nd	nd
J18	French dry-type pork sausage	0	0	2	nd	nd
J54	French dry-type pork sausage	0	1	1	nd	nd
J64	French dry-type pork sausage	3	0	1	nd	nd
LK-145	Japanese sake cellar (Japan)	0	1	3	nd	nd
LZ217	Fermented vegetables (China)	1	0	0	nd	nd
MBEL1397	Kimchi (South Korea)	0	0	2	nd	nd
MFPB16A1401	Beef carpaccio	1	0	1	nd	nd
MFPB19	Beef carpaccio	1	0	0	nd	nd
ob4.1	Human feces (South Korea)	1	1	2	nd	nd
Probio65	Kimchi	2	0	1	nd	nd
TMW 1.2	Fermented sausage (Spain)	0	0	1	–	–
TMW 1.3	Fermented sausage (Spain)	0	0	2	–	–
TMW 1.4	Fermented sausage (Spain)	nd	nd	nd	–	–
TMW 1.13	Starter culture (Germany)	nd	nd	nd	–	–
TMW 1.23	Fermented sausage (Germany)	1	0	0	(+)	+
TMW 1.46	Starter culture (Germany)	2	1	0	+	+
TMW 1.114	Starter culture (Germany)	0	1	3	–	–
TMW 1.411	Sauerkraut (Germany)	1	0	0	–	–
TMW 1.417	Starter culture (Germany)	0	1	2	–	–
TMW 1.1239	Wheat sourdough (France)	0	1	0	–	–
TMW 1.1290	Fermented sausage (Germany)	1	0	0	+	+
TMW 1.1383	Starter culture (Germany)	nd	nd	nd	–	–
TMW 1.1385	Starter culture (Germany)	nd	nd	nd	–	–
TMW 1.1386	Starter culture (Germany)	1	0	1	+	+
TMW 1.1388	Starter culture (Germany)	nd	nd	nd	–	–
TMW 1.1392	Starter culture (Germany)	nd	nd	nd	–	–
TMW 1.1393	Starter culture (Germany)	1	0	0	+	+
TMW 1.1396	Starter culture (Germany)	0	1	2	–	–
TMW 1.1397	Starter culture (Germany)	1	0	1	(+)	+
TMW 1.1398	Starter culture (Germany)	1	0	4	+	+
TMW 1.1500	Fermented sausage (Germany)	0	0	1	–	–
TMW 1.2292	Prophage free derivative of TMW 1.1398 (Janßen 2019)	nd	nd	nd	–	–
WiKim0063	Water (South Korea)	1	0	0	nd	nd
WiKim0072	Kimchi (South Korea)	1	0	0	nd	nd
WiKim0073	Kimchi (South Korea)	0	0	3	nd	nd
WiKim0074	Kimchi (South Korea)	0	1	3	nd	nd

^a^*nd* no genome/strain available

The total amount of detected prophage genomes, including their completeness evaluation, are listed in Table 1. The analysis revealed, that all *L. sakei* genomes contained prophage sequences (intact, questionable or incomplete). Twenty-one strains contained intact prophage sequences, 17 of which only harboured one, three strains harboured two and only one strain harboured three as intact predicted prophage sequences. Eleven strains contain questionable prophage sequences (one per strain) of which only two harbour additionally intact prophages (strains ob4.1 and TMW 1.46). Thirteen strains harboured only as incomplete predicted prophage sequences. In total, 26 intact, 11 questionable and 52 incomplete prophages were predicted and evaluated by PHASTER.

**Fig. 1:**
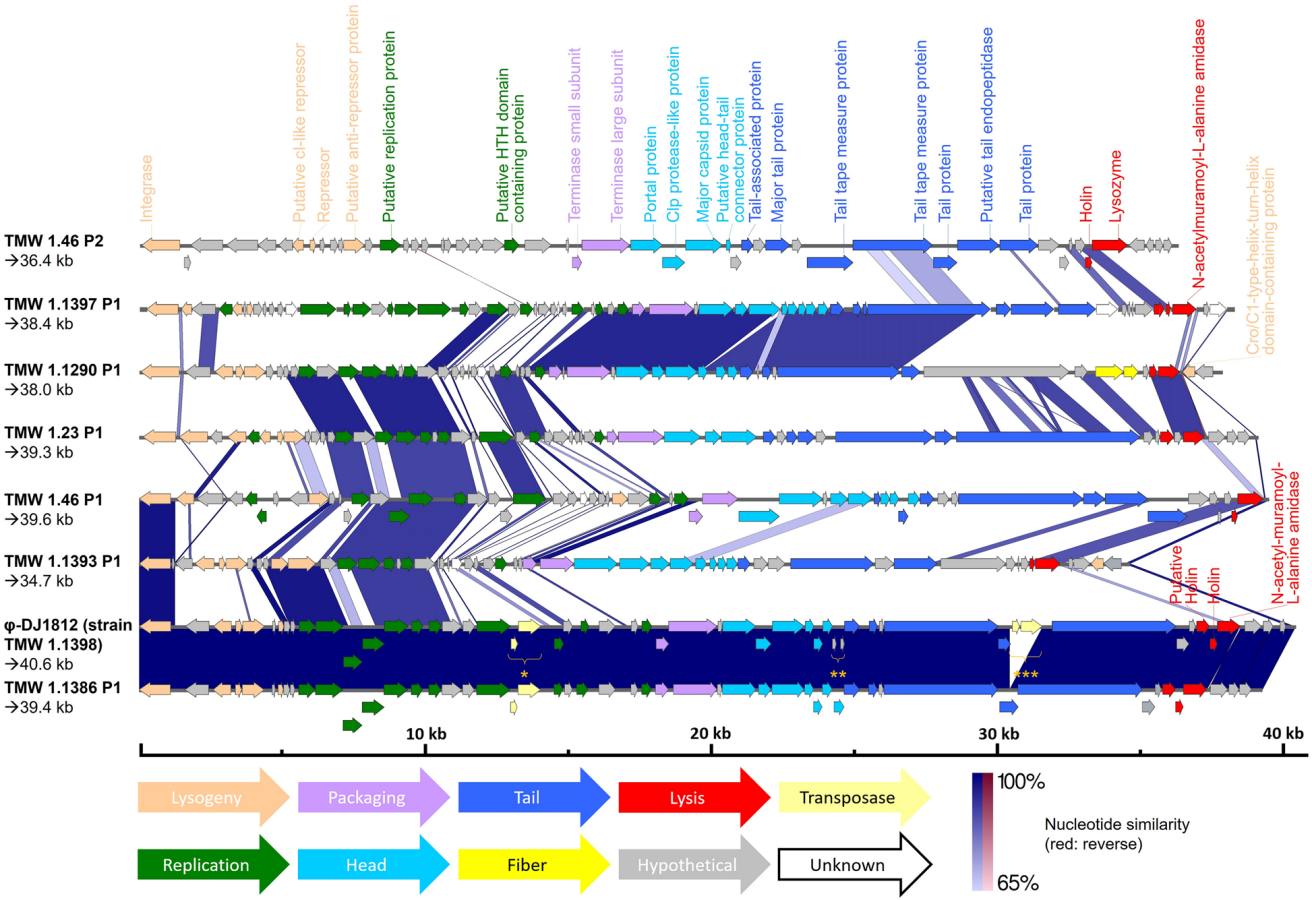
Prophage genomes with ORFs (represented as arrows) detected by PHASTER [11, 12]. Gene modules colourised after predicted task: Lysogeny (ochre), replication (green), packaging (lilac), head (light blue), tail (dark blue), fiber (yellow), lysis (red), hypothetical proteins (grey), transposase (light yellow) and phage-related proteins with unknown task (white). “*”: First transposase of phage φ-DJ1812 (harboured by strain TMW 1.1398), which is the reason for the failed contig assembly within phage TMW 1.1386 P1 after bacterial genome sequencing. “**”: SNP resulting in an incomplete ORF (compare TMW 1.1386 P1). “***”: Second transposase, which was discussed to be responsible for incomplete phage assembly of φ-DJ1812 [8]. Nucleotide similarity of 65–100% is depicted as blue bars (red if reverse similarity is given)

With exception of two prophage pairs, the pairwise comparison of those intact prophages revealed a diverse set of phages. Phage φ-DJ1812 (harboured by strain TMW 1.1398) shared an average nucleotide identity (ANI) of 99.99% over an aligned percentage (AP) of 96.26% with TMW 1.1386 P1, E28G P1 shared an ANI of 99.98% over an AP of 98.60% with MFPB19 P1 (Additional File A1). Notably, after sequencing and assembly of the genome of strain TMW 1.1386 the prophage was split in two parts (joined for the interest of this analysis), as the first transposase within TMW 1.1386 P1 was not sequenced (Fig. 1; marked with “*”). The missing sequence information was obtained later after sequencing of the viral DNA (extracted from virions) within post-induction lysates and used to complete Fig. 1. The as intact predicted prophage of strain TMW 1.411 was excluded in this analysis due to large gaps in its lysogeny and replication modules (most likely caused by an incomplete genome assembly).

### Prophage genome comparison

The as intact predicted prophage genomes by PHASTER vary in size from 33.1 kb to 55.0 kb and have GC-contents of 39–42% (compare Table 2), which is similar to the GC-contents of their hosts. The number of ORFs detected via PHASTER range from 47 (J54 P1) to 66 (CBA3635 P1). The proportion of ORFs where a likely gene function could not be assigned ranged between 16.4% (FAM18311 P1) to 58.8% (TMW 1.46 P2).

**Table 2:** General information about putatively intact *Latilactobacillus sakei* prophages. For this, prophages with the “questionable” completeness evaluation by PHASTER have been included, if all essential modules were present (checked manually). For the inducible *Latilactobacillus sakei* phages within this study, GC-content, ORFs total, ORFs with unknown function, ORF-% unknown and prophage length were adjusted after manual BLASTs. For all other as intact expected phage sequences, the values obtained by PHASTER were listed

Inducible *Latilactobacillus sakei* phages within this study:									
Phage	Predicted region by PHASTER (if multiple)	GC-content [%]	ORFs total	ORFs with unknown function	ORF-% unknown	Prophage length [kb]	Completeness (score) by PHASTER	Region position by PHASTER	Most common phage (gene hits) by PHASTER
TMW 1.23 P1		39.9	52	24	46.2	39.3	Intact (100)	contig 17: 1888–39,112	PHAGE_Lister_2389_NC_003291 (12)
TMW 1.46 P1		40.8	57	25	43.9	39.6	Intact (150)	852,024–891,679	PHAGE_Lactob_CL1_NC_028888 (7)
TMW 1.46 P2 ^a^		40.1	51	30	58.8	36.4	Intact (100)	1,197,847–1,227,163	PHAGE_Lister_B025_NC_009812 (9)
TMW 1.1290 P1		40.2	54	25	46.3	38.0	Intact (130)	contig 1: 45015–93,497	PHAGE_Lactob_PL_1_NC_022757 (10)
TMW 1.1393 P1		40.6	54	26	48.1	34.7	Intact (150)	contig 5: 69791–108,578	PHAGE_Lactob_phig1e_NC_004305 (9)
TMW 1.1397 P1		39.0	62	21	33.9	38.4	Intact (150)	contig 1: 442601–480,500	PHAGE_Lactob_Lrm1_NC_011104(8)
φ-DJ1812 ^b^ (strain TMW 1.1398)	region 1 region 2	39.3	54	18	33.3	40.6	Incomplete (50) Intact (130)	774,846–805,244 805,573–828,066	PHAGE_Brocho_NF5_NC_015252 (2) PHAGE_Lactob_LF1_NC_019486 (7)
Other putatively intact *Latilactobacillus sakei* phages:									
C21B P1		41.4	58	12	20.7	44.8	Intact (150)	346,775–391,586	PHAGE_Lactob_A2_NC_004112 (8)
CBA3635 P1		39.6	66	22	33.3	43.3	Intact (140)	239,719–283,028	PHAGE_Oenoco_phiS13_NC_023560 (16)
CBA3635 P2		39.7	53	13	24.5	51.3	Intact (150)	1,672,801–1,724,115	PHAGE_Lister_B025_NC_009812 (10)
DS4 P1 ^c^		39.8	58	19	32.8	41.2	Intact (150)	645,922–687,133	PHAGE_Entero_EFC_1_NC_025453 (4)
FAM18311 P1^c^		40.5	55	9	16.4	55.0	Intact (120)	1,267,830–1,322,910	PHAGE_Oenoco_phiS13_NC_023560 (14)
J54 P1		42.0	47	10	21.3	33.1	Questionable (70)	614,556–647,708	PHAGE_Lactoc_50101_NC_031040 (15)
J64 P1 ^c^		39.7	53	11	20.8	39.8	Intact (150)	648,583–688,460	PHAGE_Lactob_PL_1_NC_022757 (8)
J64 P2 ^c^		40.7	54	13	24.1	40.1	Intact (150)	887,011–927,148	PHAGE_Lactob_phig1e_NC_004305 (9)
J64 P3 ^a^		40.5	36	10	27.8	30.4	Intact (120)	1,294,505–1,324,922	PHAGE_Strept_315.4_NC_004587 (7)
J112 P1 ^b,3^	region 1 region 2	39.0 42.5	35 20	10 2	28.6 10.0	31.4 17.6	Incomplete (50) Questionable (80)	784,685–816,113 807,012–824,611	PHAGE_Lister_LP_030_3_NC_024384 (2) PHAGE_Lactob_CL1_NC_028888 (5)
LK-145 P1 ^c^		39.5	54	11	20.4	39.1	Questionable (70)	457,907–497,029	PHAGE_Lactob_PLE2_NC_031036 (10)
LZ217 P1 ^c^		40.4	52	12	23.1	42.7	Intact (150)	941,734–984,525	PHAGE_Lactob_phig1e_NC_004305 (8)
MFPB16A1401 P1 ^c^		40.4	51	12	23.5	37.1	Intact(150)	654,201–691,310	PHAGE_Lactob_phig1e_NC_004305 (10)
MFPB19 P1 ^c^		39.6	48	10	20.8	37.6	Intact(150)	670,108–707,803	PHAGE_Lactob_Lrm1_NC_011104 (9)
ob4.1 P1 ^a,2^	region 1 region 2	37.7 40.3	28 19	11 0	39.3 0.0	20.2 16.5	Incomplete (20) Questionable (70)	128,217–148,461 148,791–165,294	PHAGE_Lister_B054_NC_009813 (3) PHAGE_Oenoco_phi9805_NC_023559 (16)
ob4.1 P2 ^b^	region 3 region 4	40.5 39.7	23 28	0 8	0.0 28.6	19.9 27.5	Intact (130) Incomplete (10)	1,509,399–1,529,314 1,522,108–1,549,633	PHAGE_Lactob_LF1_NC_019486 (7) PHAGE_Lactob_Lrm1_NC_011104 (4)
Probio65 P1 ^c,d^	region 3	40.4	90	27	30.0	58.8	Intact (150)	1,994,946–2,053,762	PHAGE_Lactob_phig1e_NC_004305 (9)
Probio65 P2 ^c,d^	region 1 region 3	–	–	–	–	–	–	–	–
TMW 1.411 P1 ^c,e^		40.4	24	5	20.8	18.6	Intact (110)	QOSE01000002: 492–19,135	PHAGE_Lactob_PL_1_NC_022757 (7)
WiKim0063 P1 ^c^		40.2	52	12	23.1	39.2	Intact (150)	355,716–394,962	PHAGE_Lactob_phig1e_NC_004305 (10)
WiKim0072 P1 ^c^		40.7	60	16	26.7	41.9	Intact (150)	1,659,755–1,701,753	PHAGE_Lactob_phig1e_NC_004305 (10)

^a^: Phage had proximal parts not detected by PHASTER, indicating a novel phage and leading to lower completeness scores assigned by PHASTER [12]

^b^: Phage was predicted as two or more (incomplete or questionable) phage fragments by PHASTER, putatively due to BLAST hit paucity [12]

^c^: Phage was already as intact predicted in [7]. The labeling of as intact predicted phages in both studies is mostly identical. An exception is phage FAM18311 P1 (this study), which corresponds to phage FAM18311P2 of [7]

^d^: The genome of *Latilactobacillus sakei* strain Probio65 is commented by the authors as not circularized and to putatively contain a gap at the end. We found two identical prophage sequences at the proximal ends of the genome with the second one being halfed with one fragment on each proximal end of the chromosome. This may be an artefact during assembly, so we listed and counted the second prophage sequence for completeness, but excluded it from later analyses. The here as P1 listed and as intact predicted prophage by PHASTER included the first half of the second prophage sequence, artificially increasing its length and ORF count

^e^: Albeit this phage was listed as intact by PHASTER, it was missing its lysogeny module and most of the replication module. The phage showed neither inducibility by UV light nor mitomycin C during the screening. The predicted sequence was located on a proximal end of a genome contig, so it might be possible, that those missing modules are located on other contigs, yet, with exception of its putative integrase gene, located at a proximal end of a contig as well, they could not be identified (neither by alignments nor manually)

Albeit the genome organization of prophages within *L. sakei* is described at the example of prophages harboured by inducible lysogens within this study (Fig. 1), all other *sakei* prophages (Table 2) follow this type of module composition. All necessary gene modules for fully functional phage particles were present in each of those analysed prophages. These prophage genomes share a highly conserved nature in which their gene modules are arranged, similar to the previously described prophage φ-DJ1812 of strain TMW 1.1398 [8]. Yet, higher grades of nucleotide similarities (above 65%) were mainly found in the replication gene module (compare Fig. 1).

The first gene module in each prophage genome encodes genes for lysogeny and is followed by modules for replication, packaging, head, tail and fiber construction with lysis related genes at the end. Baseplate genes and most tail fibre genes (as well as host receptor binding genes) were classified as tail related genes due to ambiguous blast results. Representative examples of each gene module are listed in Table S1 (see additional material). All lysis cassettes contained a combination of one lysin and at least one holin (up to two holin-like sequences in case of TMW 1.1397 P1 and TMW 1.1386 P1 / φ-DJ1812), colourised in red. The prevalence of cell wall degrading enzymes like N-acetylmuramoyl-L-alanine amidases was expected in prophages harboured by lysogens showing inducible lysis during the screening, as they are essential for host lysis.

### Integration sites of intact phages

To analyse, if the as intact predicted phages favor specific genomic loci for integration, the prophage harbouring bacterial genome regions were aligned to the reference genome of *L. sakei* subsp. *sakei* 23 K (CR936503.1). Additionally, attachment sites were determined/checked by BLASTing the proximal ends of each prophage (with a bacterial genome overlap of a view thousand base pairs) against one another. Albeit aligning the integration locus of *L. sakei* TMW 1.23 P1 by using the bacterial genome of strain TMW 1.23 was not possible, as the prophage occupied a whole genome contig with no bacterial border regions, putative attachment sites were found through the phylogenetic analysis of its integrase gene, hinting at an integration in a tmRNA gene (for further explanation see chapter “Phylogenetic analysis“). The integration sites, including both attachment sites of each as intact predicted phage, are listed in Table 3.

**Table 3:** Integration sites of as intact predicted sakei phages. Listed are the attachment sites attL and attR of each phage, as well as the gene products and the locus tags of the genes where those phages insert

Phage	attL (Integrase site)	attR (Lysin site)	Insertion site (gene product)	Locus tag
TMW 1.1290 P1	TACCCTACGGACCCTTCCA	TATCCTACAGACCCTTCCA	Fe-S cluster assembly protein SufB	KNO49_00270
TMW 1.46 P2	TATCCGACGGAGCCTTCCA	TAGCCCACGGACCCTTCCA	Fe-S cluster assembly protein SufB	A4W82_06315
J64 P3 (region 4)	TTATAGGCGTTCGTTTAATT	TTATAGACGTTCATTTAATT	Glucose-6-phosphate isomerase	LSAJ64_RS06935
CBA3635 P1	TCTATTCCCATTCCACTGTT	TCTATTCCCATTCAATTGTT	Glutamine-hydrolyzing GMP synthase	H3M14_RS01450
ob4.1 P1	AACAGTGGAATGGGAATAGA	AACAATTGAATGGGAATAGA	Glutamine-hydrolyzing GMP synthase	KIK01_RS00640
J112 P1	AATTTGACCATAATTAATTACC	AATTTGACCATAATTTAAAACC	Hypothetical protein	LSAJ112_RS03875
J64 P2 (region 3)	AATTTGACCATAATTAATTACC	AATTTGACCATAATTTAAAACC	Hypothetical protein	LSAJ64_RS04570
LZ217 P1	AATTATGGTCAAATT	AATTATGGTCAAATT	Hypothetical protein	CFK76_RS05020
Probio65 P1	AATTTGACCATAATT	AATTTGACCATAATT	Hypothetical protein	LP065_RS09960
TMW 1.1386 P1	AATTTGACCATAATTAATTACC	AATTTGACCATAATTTAAAACC	Hypothetical protein	KNO52_05825
TMW 1.1393 P1	AATTTGACCATAATTAATTACC	AATTTGACCATAATTTAAAACC	Hypothetical protein	KNO57_04740
TMW 1.46 P1	AATTTGACCATAATTAATTACC	AATTTGACCATAATTTAAAACC	Hypothetical protein	A4W82_04310
WiKim0063 P1	AATTTGACCATAATTAATTACC	AATTTGACCATAATTTAAAACC	Hypothetical protein	LBS_RS02005
WiKim0072 P1	AATTTGACCATAATTAATTACC	AATTTGACCATAATTTAAAACC	Hypothetical protein	CW750_RS08280
φ-DJ1812 (strain TMW 1.1398)	AATTTGACCATAATTAATTACC	AATTTGACCATAATTTAAAACC	Hypothetical protein	A4W88_04025
TMW 1.23 P1	AATGGAGCCGGCG	AATGGAGCCGGCG	ssrA (transfer-messenger RNA)	KNO63_02330
FAM18311 P1	AATGGAGCCGGCG	AATGGAGCCGGCG	ssrA (transfer-messenger RNA)	B4V05_RS06785
C21B P1	TATGCGCCACCCGGGA	TATGCGCCACCCGGGA	tRNA-Arg	FXV74_RS02010
CBA3635 P2	TCCCGGGTGGCGCATA	TCCCGGGTGGCGCATA	tRNA-Arg	H3M14_RS08475
DS4 P1	TATATGCGCCACCCGGGA	TATATGCGCCACCCGGGA	tRNA-Arg	C0213_RS03410
E28G P1	TAAAATGTCACAGGCG	TAAAATGTCACAGGCG	tRNA-Arg	FX990_RS02215
J54 P1	ATGCGCCACCCGGGAGT	ATGCGCCACCCGGGAGT	tRNA-Arg	LSAJ54_RS03305
J64 P1	ATTATGCGCCACCCGGGA	ATTATGCGCCACCCGGGA	tRNA-Arg	LSAJ64_RS03525
MFPB16A1401 P1	TATGCGCCACCCGGGAGT	TATGCGCCACCCGGGAGT	tRNA-Arg	MFPB16_RS03465
MFPB19 P1	TAAAATGTCACAGGCG	TAAAATGTCACAGGCG	tRNA-Arg	MFPB19_RS03605
TMW 1.411 P1	TAAAATGTCACAGGCG	TAAAATGTCACAGGCG	tRNA-Arg	DT321_01900
LK-145 P1	CCTGCCACGGGCAT	CCTGCCACGGGCAT	tRNA-Leu	CCX78_RS02335
ob4.1 P2	ATGCCCGTGGCAGG	ATGCCCGTGGCAGG	tRNA-Leu	KIK01_RS07950
TMW 1.1397 P1	ATGCCCGTGGCAGG	ATGCCCGTGGCAGG	tRNA-Leu	NCX38_RS02535

The integration analysis highlighted six chromosomal integration sites shared by all as intact predicted phages. Most of these inserted either in arginine (nine phages) or leucine (three phages) tRNA genes or in the same gene with an unknown function (ten phages), yet some prophages were found in genes coding for a tmRNA (two phages), a glutamine-hydrolysing GMP synthase (two phages), a *sufB*-like gene (two phages), which is part of an iron-sulfur cluster synthesis operon and a Glucose-6-phosphate isomerase (one phage).

### Prophage inducibility

**Fig. 2:**
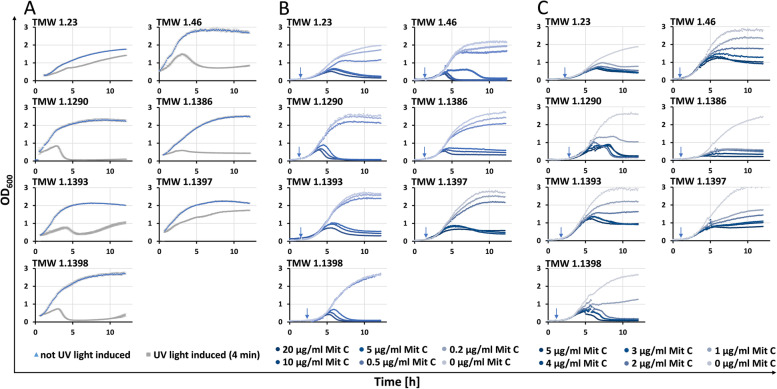
Growth curves of *L. sakei* strains with inducible lysis after induction. UV light induction performed shortly before transferring samples into reader. Time of mitomycin C induction is marked with an arrow. Y-axis: Turbidity measured at 600 nm (scale: 0–3). X-axis: Time in hours (scale: 0–14). A: Induction with UV light for 4 min and without UV light induction. B: Induction with different amounts of mitomycin C (20 μg/ml, 10 μg/ml, 5 μg/ml, 0.5 μg/ml, 0.2 μg/ml, 0 μg/ml). C: Induction with different amounts of mitomycin C (5 μg/ml, 4 μg/ml, 3 μg/ml, 2 μg/ml, 1 μg/ml, 0 μg/ml)

In order to identify new lysogens and to determine, if strains with as intact predicted prophage sequences can be induced, induction experiments were performed. Therefore, UV light as well as mitomycin C were used. Lysis was monitored by a temporary halt (weak lysis), or rapid decrease (strong lysis) of OD_600_ in growth curves after induction (Fig. 2 A - C).

Preliminary experiments have shown, that the lysis responses of induced phages are strongly dependent on their growth phase at the time of induction and the amount of UV light or mitomycin C utilised. Too early induction (with excessive usage of UV light or mitomycin C) hampered growth until lysis was undetectable, after too late induction lysis occurred not any more. Significant results were obtained after approximately 2 hours of bacterial growth (Start OD_600_ ≈ 0.1, Induction OD_600_ ≈ 0.3–0.6) for a four-minute UV light induction and doubling of OD_600_ (Start OD_600_ ≈ 0.05, Induction OD_600_ ≈ 0.1; time depending on used strain) for induction with different amounts of mitomycin C. Lysis occurred approximately 2–3 h after UV induction and 3–4 h after mitomycin C induction, depending on the mitomycin C concentration used. Mitomycin C concentrations of 0.2 and 0.5 μg/ml within the screening had only minor effects on the growth of *L. sakei* within the screening, whereas higher concentrations hampered growth (5 μg/ml and above), as was expected. Lytic *sakei* strains (and thus potential lysogens) where induced again in a separate experiment, using mitomycin C concentrations ranging from 1 to 5 μg/ml to optimise phage induction and reduce the toxic effect of mitomycin C on the bacterial cells (Fig. 2 C). Overall, twenty-four *L. sakei* strains were screened for their prophage inducibility (Table 1). Seven strains showed inducible lysis after UV light or mitomycin C treatment, two of which (TMW 1.46 and TMW 1.1398) already had genomic data available and intact prophages were predicted. The growth curves of the seven inducible *L. sakei* strains after UV light (A) and mitomycin C (B and C) treatment are displayed in Fig. 2. Growth curves of strains classified as non-inducible after mitomycin C treatment are summarized in Additional File A2.

Only strains with as intact predicted prophages showed strongly pronounced lysis, verifying that host lysis is directly connected to the prevalence of fully functional prophages, considering those strains lacked other remnant phages (in case of strains TMW 1.23, TMW 1.1290, TMW 1.1386, TMW 1.1393 and TMW 1.1397) or harbour remnant phages without lysins (strains TMW 1.46 and TMW 1.1398) which could theoretically affect lysis. Strains with UV light inducible prophages, also coherently display lysis after induction via mitomycin C (see Fig. 2 B and C). Each inducible strain with exception of TMW 1.46, harbouring two individual intact prophages, had only one detected intact prophage. This simplifies assigning the lysis response to a specific prophage element (albeit lysis caused/enhanced by autolysins might still occur to some extent and cannot be dismissed). Strain TMW 1.46 showed strong reproducible lysis after UV light induction but was difficult to induce with mitomycin C. The strain showed a delayed lysis in response to an induction with 5 μg/ml mitomycin C (see Fig. 2 B) during the screening. The reason for this is still unknown, yet we speculate higher amounts of mitomycin C are needed for strong reproducible phage-mediated lysis of strain TMW 1.46. This was confirmed after mostly lower mitomycin C concentrations (1–5 μg/ml) were tested (Fig. 2 C), leading to considerably weaker lysis responses. Induction with 5 μg/ml mitomycin C as control showed here also less lysis, putatively a sign for impaired reproducibility when less of the antibiotic was used. Both prophages of strain TMW 1.46 showed all essential gene modules for functional phages, including lysis genes coding for a holin and a lysin, as seen in Fig. 1. It is not clear yet, which of the prophage sequences (or a combination thereof) caused the strain to lyse, albeit both phage virions could be detected by PCR in the lysates 24 h after induction and viral DNA of both virions could be isolated from purified lysates.

**Fig. 3:**
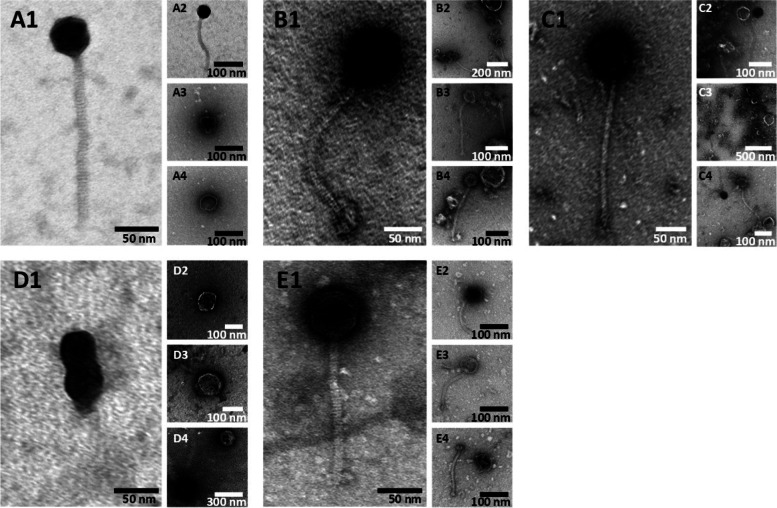
Electron micrographs of purified and negative stained lysates of UV light induced *Latilactobacillus sakei* strains harbouring at least one as intact predicted prophage after the lysis event. A1-A4: *L. s.* TMW 1.23, B1-B4: *L. s.* TMW 1.46, C1-C4: *L. s.* TMW 1.1290, D1-D4: *L. s.* TMW 1.1386, E1-E4: *L. s.* TMW 1.1393

Strain TMW 1.23 showed only a weak lysis response to UV light (more pronounced after mitomycin C treatment). Nonetheless, after whole genome sequencing and the PHASTER analysis an intact prophage could be detected (Fig. 1), resulting in a seemingly fully assembled phage particle as verified by TEM (see Fig. 3 A1 - A2).

Strain TMW 1.1393 showed strong growth regeneration after the UV induced lysis event, represented by a fast increase of turbidity directly after the minimal cell density was reached (approximately 6 hours after start of the OD-measurement). This putatively was caused by an insufficient induction treatment, resulting in overgrowth of the UV light damaged cells by uninhibited ones. The growth curves after mitomycin C induction showed no regeneration, indicating that mitomycin C might be in this case the more efficient induction method. Nonetheless, in previous works *Lactococcus lactis* TIFN1 showed a similar growth regeneration after induction with 1.5 μg/ml mitomycin C at a higher initial and induction-OD_600_ (start-OD_600_ = 0.1; induction-OD_600_ = 0.2) [13], emphasizing the importance of optimally chosen induction parameters.

As previously mentioned, the intact prophages of the strains TMW 1.1386 and TMW 1.1398 only differ in one transposase (Fig. 1; marked with “***”). Yet, TMW 1.1398 showed a higher decrease in turbidity during the lysis event, suggesting that induced lysis is also influenced by the strain in which the prophage is located.

To gain insights into the virion morphology and to verify successful prophage induction, the lysates of five UV induced strains containing at least one previously as intact predicted prophage were harvested after the lysis event, purified, negatively stained, and subjected to transmission electron microscopy (electron micrographs depicted in Fig. 3 A - E; A1 - A4: Strain TMW 1.23; B1 - B4: Strain TMW 1.46; C1 - C4: Strain TMW 1.1290; D1 - D4: Strain TMW 1.1386; E1 - E4: Strain TMW 1.1393).

Excluding the lysate of strain TMW 1.1386 (Fig. 3 D1 - D4), all lysates contained seemingly fully assembled phage particles with icosahedral heads of approximately 50 nm in diameter and long, most likely non-contractile tails (as no retracted sheath tubes like seen in myovirus micrographs [14] were present in the micrographs) of different lengths, suggesting an affiliation to the siphovirus morphotype. Nonetheless, in the micrographs of strains with seemingly intact phage progeny empty headed virions (e.g. Figure 3 E3) could be found as well as separated heads and/or tails (e.g. Figure 3 A3 - A4, Fig. 3 E4). In contrast, purified TMW 1.1386 post-induction lysates only showed unshapely, head-like structures in the range of 50 to 200 nm in size and no preassembled or attached tails were detectable in the micrographs (Fig. 3 D1 - D4).

Interestingly, in lysates of UV light induced strain TMW 1.46 only one virion morphotype was found (Fig. 3 B1 - B4), albeit the presence of two as intact predicted, potentially inducible prophages.

To analyse, if both prophages within *L. sakei* TMW 1.46 are inducible and able to form virions post induction, PCR experiments were performed. In the first experiment, the ability to circularize without prior induction, and hence spontaneous phage induction, was confirmed for both prophages (Fig. S1 in the additional material). In a second experiment the sterile filtered lysate of UV light induced *L. sakei* TMW 1.46 (obtained 24 h after induction) was freed from any bacterial DNA through digestion with DNase I, followed by PCR only amplifying viral DNA protected previously by viral capsids. The amplification of both prophage markers (Fig. S2 in the additional material) agreed with our assumption, that both as intact predicted prophages are indeed inducible and able to form virions.

As an additional proof for successful induction next to transmission electron micrsoscopy, we extracted and sequenced viral DNA from purified lysates of all lytic lysogens. We found viral DNA in all samples, albeit some phage genomes were only partially sequenced (TMW 1.1290 P1 and TMW 1.1398 P1). TMW 1.23 P1 was sequenced as a prophage, including adjacent bacterial genome parts, confirming our hypothesis of this prophage integrating in a tmRNA gene (see chapter integration site analysis). The small library after sequencing and low genome coverage (data not shown) of phage TMW 1.23 P1 both confirm, that only few virions were released after (weak) host lysis. Three of the obtained viral sequences after assembly were marked as circular (TMW 1.1386 P1, TMW 1.1393 P1, TMW 1.1397 P1). The sequencing data of TMW 1.46 P1 and P2 after assembly consisted of three contigs (contig 1 contained phage most of TMW 1.46 P1, contig 2 contained most of phage TMW 1.46 P2, and contig 3 contained the 227 bp long, for both phage genomes identical, missing tail gene part needed for full coverage and circularity), indicating the successful induction of both prophages within this strain. Circularity was proven exemplary for the viral DNA of phage TMW 1.1393 P1, as all expected fragments (fragment lengths including overhang: 306, 327, 395, 567, 1876, 2091, 2238, 3561, 4000, 4326, 4396, 5126, 5558) were present after digestion via EcoRI (compare agarose gel in Additional File A3). In all sequenced, circular phage genomes (including TMW 1.46 P1, TMW 1.46 P2, TMW 1.1386 P1, TMW 1.1393 P1 and TMW 1.1397 P1) the attL-site was present, but the attR-site was missing (compare Table 3).

### Phylogenetic analysis

To obtain more information about genetic diversity of the newly found temperate phages, three phylogenetic trees were created with marker genes of different genetic modules (integrase, terminase (large subunit) and tape measure protein (TMP; the first sequence was used if multiples were available)). As outgroups respective genes of other LAB-phages (see section “Availability of data and materials”) were used.

The phylogenetic analysis reproduced the previously mentioned high sequence similarities of TMW 1.1386 P1 and φ-DJ1812 (strain TMW 1.1398) [8], as well as of E28G P1 and MFPB19 P1.

**Fig. 4:**
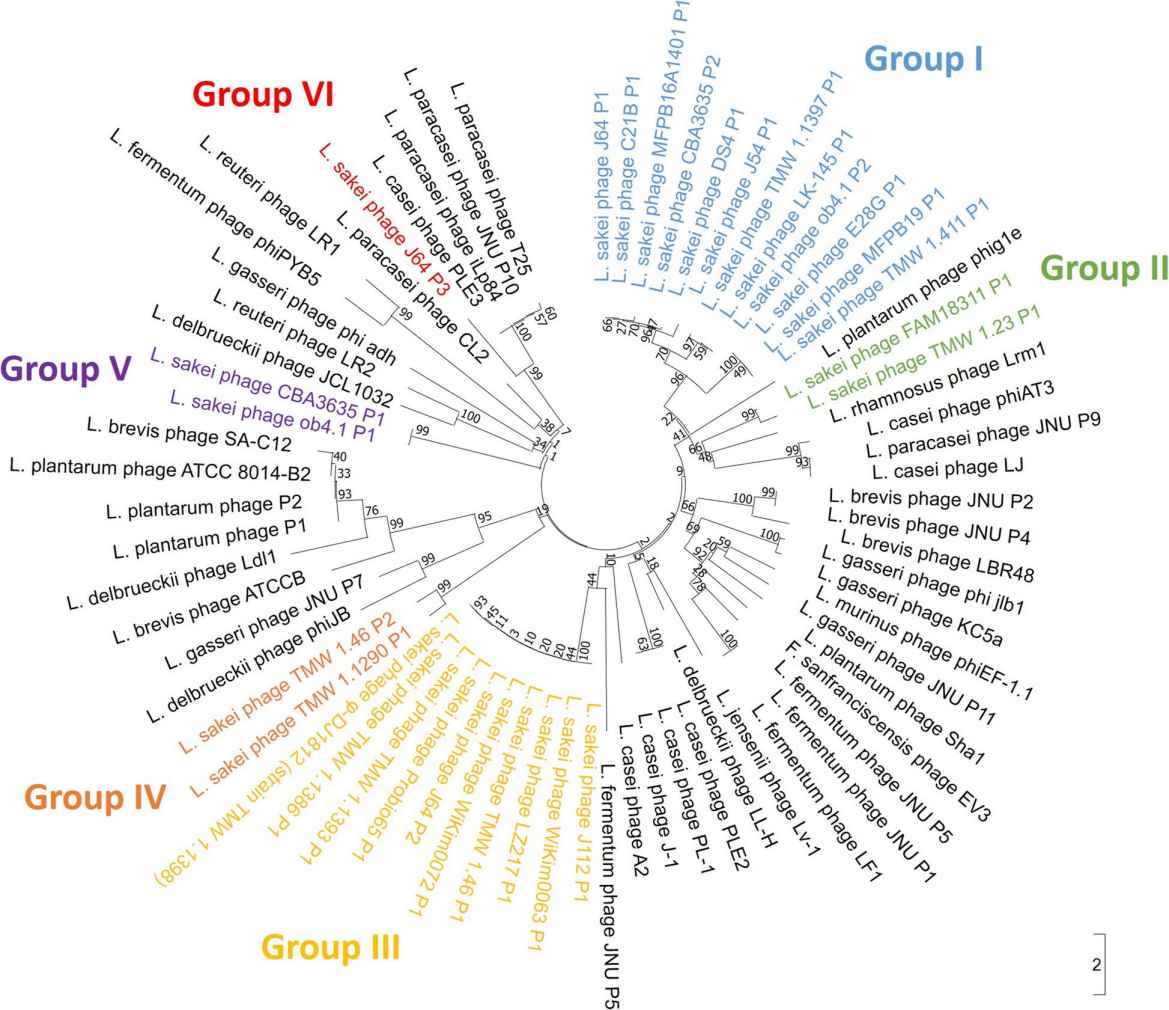
Neighbor joining tree of the phage integrase gene of as intact predicted prophages in *L. sakei* strains. Bootstrap values are based on a Jukes-Cantor model (1000 replicates). The integrases of phages infecting other lactobacilli were included as outgroups. For the assignment of groups based on the genomic integration sites, only *Latilactobacillus sakei* phage integrases have been taken into account

A close phylogenetic relationship of phage integrases (Fig. 4) is reflected by the identical integration sites into the genomes of their bacterial host (Table 3). Therefore, different groups have been introduced which reflect the clustering/sharing of the same integration site. Notably, only *sakei* phages have been taken into account for the assignment into these groups.

Phage members of group I insert in arginine and leucine tRNA genes and share, as visualised in the respective phylogenetic tree (Fig. 4), similar integrases with percent identities ranging from 43.58 to 100.00% (Additional File A1).

Due to before mentioned reasons (prophage occupies a whole contig), the integration site of TMW 1.23 P1 could first not be determined via alignment onto a reference genome. Its integrase however, shares 69,16% identity with the integrase of Latilactobacillus sakei phage FAM18311 P1 (Additional File A1), thus we hypothesised the same genomic integration site in a tmRNA gene might be possible. This hypothesis was verified after sequencing of the viral DNA. The obtained sequence data contained, most likely due to low DNA amounts extractable from the purified lysates of this strain, the prophage sequence with adjacent bacterial genome sequences making the alignment possible. Therefore, both phages were assigned to group II (Fig. 4).

The phages inserting in the same gene with unknown function (group III; Fig. 4), share high percent identities ranging from 96.89 to 100.00% in the pairwise comparison (Additional File A1). Group IV phages inserted into a SufB coding gene and shared integrases with 61.63% percent identity. Group V phages, inserting in a gene coding for a glutamine-hydrolysing GMP synthase, share integrases with a percent identity of 96.30%. Only one *sakei* prophage was integrated in a gene coding for a glucose-6-phosphate isomerase and was therefore solely assigned to group VI. Its integrase gene shares only 27.92 to 36.79% percent identity with all other integrases of found *sakei* prophages.

In the phylogenetic trees constructed with the terminase (large subunit) and tape measure protein chosen as marker genes (Figs. S3 and S4 in the additional material), the outgroups (phages infecting other lactobacilli) clustered in between *sakei* phages as well, suggesting high levels of diversity between those groups. Some of the phage groups (especially group I and III; Fig. 4) cluster also partially together (and sometimes mix) in the other phylogenetic trees, whereas the topology of the *sakei* phages within the trees mostly changes, depending on the selected marker gene and under consideration of before mentioned caveats.

## Discussion

The predictive genomic analysis via PHASTER revealed remnant, or intact prophages in all 43 analysed *L. sakei* genomes. This suggests each of those strains were attacked by phage in the past. This is in accordance with a previous study, which analysed (pro)phages in *Levilactobacillus brevis* [15]. The authors showed, that of 19 analysed *L. brevis* genomes all harboured prophage sequences of some kind (remnant or intact). Fifteen of their strains contained intact prophages (27 intact prophages in total) with one to four prophages per strain. Only four of their strains lacked intact or questionable prophage regions.

Although authors have scanned various *Lactobacillus* species, which include some of the *Latilactobacillus sakei* strains incorporated in our study, for prophages in the past and thus provided valuable data for our work to compare to, e.g. the suggestion of discrete integration sites within the analysed genomes, the resolution of their data are confined to relative genomic positions in contrast to gene level (perspicuously, considering 1472 genomes across 16 species were analysed by the authors), which would enable further hypotheses why these genes might have been chosen as integration sites [7]. To add to this data, we provided accurate, corrected attachment sites for each of our as intact predicted *sakei* prophages, which PHASTER could not often detect reliably, as well as genomic integration sites on a gene level.

Our analysis of the chromosomal prophage integration sites in *L. sakei* revealed six discrete loci. Phages using the same integration site were also found to share closely related integrase genes. This sharing of integration sites between closely related phages has also been reported in temperate phages of *L. brevis* [15].

Most of the found phage sequences inserted either in the same gene with an unknown function (group III), hindering a prediction why this site may have been chosen for integration, or in tRNA (or tmRNA) related genes (group I and II). Latter being described as common targets for phage integration via tyrosine recombinase family integrases as summarised by Williams [16].

Two of the *L. sakei* prophages (TMW 1.1290 P1 and TMW 1.46 P2; Group IV) were integrated into a *sufB*-like gene, part of an iron-sulfur cluster synthesis operon, which has been studied in *E. coli* and was proposed to play an important role during oxidative cell stress [17]. Using DNA microarray-mediated transcriptional profiling, Zheng et al. demonstrated the upregulation of this operon under oxidative stress caused by hydrogen peroxide [18], which is also known to induce some prophages [19], suggesting a connection between prophage induction and the iron-sulfur cluster biosynthesis pathway. Indeed, Maynard et al. demonstrated, that this pathway grants protection during lambda phage infection [20]. This corroborates the hypothesis, that the chromosomal integration site of the two phages TMW 1.46 P2 and TMW 1.1290 P1 within this study was not chosen at random but rather intentionally provoked by the phages or their host.

The last two integration sites comprised genes coding for a glutamine-hydrolysing GMP synthase (group V) and a glucose-6-phosphate isomerase (PGI; Group VI). Albeit there is PGI-related information connected to coli phage mu available (phage inserted into a PGI-gene, hence the strain was serving as a PGI-negative mutant), this seems rather coincidental. In relation to the glutamine-hydrolysing GMP synthase as integration site, there is no literature data available. Interestingly, both of those genes seem detrimental to proper growth, as PGI is responsible for catalysing the reversible isomerisation of glucose 6-phosphate to fructose 6-phosphate during glycolysis and glutamine-hydrolysing GMP synthase plays a key role during the purine metabolism. Phages are known to restore the genes integrity in which they integrate by carrying a duplication of the interrupted gene portion with them [21]. Impairment of those respective pathways und thus growth of their host might therefore be unlikely, albeit in theory possible.

Using PHASTER to search for the prophages in *L. brevis* SA-C12 (e. g. TPSAC12–2; data not shown) mentioned by Feyereisen et al. [15], revealed the same succession of prophage gene modules displayed for the prophages of *L. sakei* within this study*.* Yet, the temperate phage genomes of the previously mentioned publication had been displayed beginning with the small subunit terminase gene, most likely to facilitate an easier comparison with genomes of virulent phages within the same study. The dissimilarities in the gene module order of prophage sequences and viral genomes (as obtained after DNA extraction and sequencing directly from purified phage particles) could arise, if the virus genomes circularize before integration, and integrate into the genomes of their bacterial host by using their attachment sites (att). These are often found in close proximity of the integrase gene as shown in coli phage lambda [22], leading to prophage sequences which then start with an integrase gene. A circularly permutated relationship between prophage and virion DNA was already reported for prophage pKO2 of *Klebsiella oxytoca* [23]. The tendency for prophage circularization (without prior induction) was proven for both prophages of *L. sakei* TMW 1.46 within this study as well as for phage φ-DJ1812 [8] via PCR. Interestingly, after extraction and sequencing of the viral DNA in post-induced, purified lysates of the lytic lysogens, some of the genomes, obtained after assembly, were marked as circular, which we prove exemplary via digestion of the viral genome of TMW 1.1393 P1 with EcoRI. We therefore can assume, that these prophages can not only circularize within the host cell to some extent without prior induction, they also lead to virions with circular genomes packed inside them after induction.

UV light and DNA damaging substances, e. g. antibiotics like ciprofloxacin or mitomycin C, can activate the cell’s SOS response by enhancing transcription of *recA* and in turn lead to amplified phage induction [24, 25]. While ciprofloxacin is forbidden in the EU for veterinary use, the usage of enrofloxacin in rabbits, chickens and turkeys has been authorised by the committee for medicinal products [26]. In vivo, enrofloxacin converts partly into its main metabolite ciprofloxacin as shown in chicken [27]. This raises the question, if starter strains used in the production of fermented meat products could come in contact with these antibiotics and thus experience enhanced phage induction, potentially leading to lower product quality. This is especially concerning if chicken meat is used for fermentation as fat reduced alternative to beef and pork in context of enrofloxacin being used in European domestication.

In this study we utilised UV light and mitomycin C to trigger the SOS response of different lysogens and thus induce harboured intact prophages as shown in previous studies [15, 24, 28, 29]. This way we found seven *L. sakei* strains with lytic behaviour after induction.

In accordance with previous studies about temperate phage in *Lactococcus lactis* [13], our preliminary experiments verified the importance of growth phase and amount/intensity of the inducer. Extreme growth (very fast or very slow growing strains) was found to be disadvantageous and might have resulted in non-detectable lysogens during the screening. We found to receive the best induction results across all tested *sakei* strains while inducing them at an OD_600_ = 0.1 for mitomycin C and an OD_600_ = 0.3 for UV light treatment.

Although phage-mediated lysis of the lysogenic *sakei* strains presented in this study could be observed at mitomycin C concentrations as low as 0.5 μg/ml (strain TMW 1.23), most of them needed higher amounts of the antibiotic to show clear signs of lytic behaviour. Within the screening and of the tested concentrations, 5 μg/ml to 20 μg/ml mitomycin C often showed the best results, although these values are high, compared to other studies using mitomycin C as prophage inducer. Usually, mitomycin C is added, due to its toxicity at higher concentrations, in much smaller amounts (0.1 μg/ml to a maximum of 2 μg/ml (final concentration)) in similar experimental settings with different LAB like miscellaneous lactobacilli [15, 30], *Lactococcus lactis* [13] and *Streptococcus pyogenes* [31].

Bacteriophages are already known for over a century, with the first publication mentioning these “ultra-microscopic viruses” in 1915 [32]. Phage classification since, first performed by Bradley in 1967 [14], heavily relied on phage morphology as modern molecular and bioinformatic tools were not available at that point of time.

To date all phages found in lactic acid bacteria belong to the former order *Caudovirales*, most of them affiliated to the former *Siphoviridae* phage family (today the siphoviruses are listed as morphotypes under the class *Caudoviricetes* [33]), characterised by isomeric, icosaedric heads and long non-contractile tails [34]. The virions detected by TEM within this study are no exception as they all showed these morphological features. Interestingly, in contrast to the abolished *Siphoviridae* family, described as having genomes composed of linear, double stranded DNA (dsDNA), the genomes extracted from virions of the induced *sakei* strains were mostly marked as circular. A similar phage (with the siphovirus morphotype and a circular dsDNA genome) was found in the *Enterococcus faecalis* phage EFC1 which belongs to the new genus *Saphexavirus* [35].

While empty or separated phage heads as well as separated tails were generally expected, as they represent important steps during siphovirus phage assembly [36], the absence of fully-assembled virions in the lysate of *L. sakei* TMW 1.1386 after UV light induction cannot be explained with certainty. As mentioned above, its prophage TMW 1.1386 P1 is nearly identical to phage φ-DJ1812 harboured by *L. sakei* TMW 1.1398, with exception of a pair of transposases in one of its tail genes, which was formerly discussed to cause an incomplete phage assembly (defective head-tail joining). The missing of these transposases in TMW 1.1386 P1 could have therefore led to fully assembled virions. Nonetheless this could not be verified by TEM. A possible explanation for this could be the weaker lysis after UV light induction (compare Fig. 2 A), followed by fewer phage particles being released and unlikely to be found by TEM. Another explanation could be the second pair of transposases in the replication gene-cluster (Fig. 1; marked with “*”) of TMW 1.1386 P1, which can also be found in the prophage of strain TMW 1.1398.

Most of the inducible lysogens within this study harbour only one as intact predicted prophage in their genome, which facilitated assigning the released phage progeny after induction to their correct coding prophage. Interestingly, only one morphotype of phage progeny was found in the lysates of *L. sakei* TMW 1.46 after induction with UV light (compare micrographs Fig. 3 B1 - B4), albeit the strain harbours two as intact predicted prophages. This is especially interesting, considering simultaneous induction of multiple prophages within the same genome, leading in turn to various phage particles observable with EM, is generally possible, as demonstrated in *Silicibacter* [37]. Furthermore, Mahony et al. have demonstrated by deleting specific regions within the TMP gene in the lactococcal phage TP901–1, which resulted in shorter tails of the produced phage progeny, that virion tail length is dependent on the length of the TMP gene [29]. Deriving from prophages with different sized TMP genes, the virions of TMW 1.46 were therefore expected to also vary in tail length.

As it was not possible via TEM to determine which of the two prophages (or a combination thereof) was induced after UV light treatment, a prophage specific PCR was performed, verifying the ability of both prophages in a non-induced cryo culture of *L. sakei* TMW 1.46 to circularize. Genome circularization is commonly seen in bacterial viruses, inter alia directly after infection [38] or during lytic development [39]. Circularization of prophage genomes by site specific recombination of both phage attachment sites without prior induction, was also reported previously in *E. coli* [40], albeit the authors showed that circularization events were strongly promoted by induction with mitomycin C. Detection of both phage markers with our second qualitative PCR approach in DNase I treated lysate of post UV light induced *L. sakei* TMW 1.46, lets us assume that both virions are produced, as the viral capsids should protect the viral DNA from DNase I digestion. Furthermore, we were able to sequence viral DNA originating from both prophages in the purified post-induction lysates of this strain, indicating the successful induction of both prophages. The distribution of phage progeny produced by *L. sakei* TMW 1.46 after induction treatment is still unknown, but might be clarified by a quantitative PCR approach in the future.

## Conclusions

In this study we screened twenty-four strains for intact prophages by inducing them via UV light or mitomycin C. Seventeen genomes of inducible and non-inducible strains included in the screening were searched for intact prophages. This revealed seven strains harbouring one to two putatively intact prophage sequences, which in turn are able to lyse their host after induction.

These temperate phages seem to insert into discrete loci within their host’s chromosomes, one of them being an iron-sulfur cluster, which was discussed to grant protection against prophages in the past. Genomic analysis of the prophage sequences revealed a highly conserved nature in how their gene modules are arranged, comparable to previous observations in other LAB. Every prophage seems to contain the full repertoire of genes needed for the assembly of intact phage virions, including a lysis cassette responsible for host lysis.

Once host lysis occurred after prophage induction, phage virions could be isolated and detected via electron microscopy. The morphological analysis of those phage particles revealed, that all of them most likely belonged to the siphovirus morphotype, commonly represented in lactic acid bacteria. Interestingly, the presence of transposases in some of the prophages might have caused an incomplete assembly, as fully assembled virions were not detectable via TEM in the corresponding lysates.

Summarised, the genome analysis, the lysis screening, as well as the EM analysis of purified phage virions obtained after prophage induction give strong evidence that temperate phage are closely connected to *L. sakei.* Albeit the impact of those prophages on the manufacturing of fermented meat products and their host’s fitness overall still has to be studied, this work is an important step to understand the incidence of temperate phage in the meat associated organism *Latilactobacillus sakei*.

## Methods

### Bacterial growth, cultures and strains

For 1 L modified De Man, Rogosa and Sharpe Medium (mMRS), 2 ml magnesium sulfate, heptahydrate (Sigma-Aldrich) stock solution (0.1 g/ml, solubilised in water) and 0.5 ml mangan(II)sulfate, monohydrate (Carl Roth) stock solution (0.1 g/ml, solubilised in water) were added to 10 g Trypton/Pepton from Casein (Carl Roth), 10 g meat extract (Millipore), 5 g yeast extract (Carl Roth), as well as 2.6 g di-potassium hydrogenphosphate, trihydrate (Merck), 8.3 g sodium acetate, trihydrate (Carl Roth), 2 g di-ammonium hydrogencitrate (Carl Roth), as well as 1 g Tween 80® (Fisher Scientific). The volume was adjusted to 900 ml with demineralised water. After setting the pH to 6.2–6.5 with 6 M hydrochloric acid, the volume was adjusted to 950 ml with demineralised water. Autoclaving was performed at 121 °C for 15 min. 22 g D-Glucose, monohydrate (Gerbu) were solved in 50 ml demineralised water and autoclaved separately. After autoclaving the medium was completed by adding the D-Glucose stock. For mMRS-Agar (mMRS(A)) an additional 15 g agar-agar (Carl Roth) was added before autoclaving.

*Latilactobacillus* strains of the in-house strain collection were plated on mMRS(A) and incubated overnight, static and at 30 °C. Of each plate 4 colonies were picked and transferred onto a fresh mMRS(A)-plate and incubated for 48 h. After species validation with MALDI-TOF, one colony was transferred into liquid mMRS in a 15 ml centrifuge tube and incubated with closed lid like mentioned above. After centrifugation the cell pellet was resuspended in 1.8 ml mMRS of which 0.9 ml was transferred into a cryo vial containing 0.9 ml sterile 80% glycerol. The cryo stocks were stored at − 80 °C.

For preparation of *Latilactobacillus sakei* overnight cultures 15 ml at room temperature (RT) preheated mMRS were inoculated with 15 μl of the formerly described cryo stocks (≙ 1:1000 dilution). The cultures were incubated overnight, static, at 30 °C, with closed lid.

*Latilactobacillus sakei* strains (listed in Table 1) were cultured at 30 °C in mMRS broth [41] modified by using the same amount of di-ammonium hydrogencitrate (Carl Roth) instead of ammonium citrate.

### Prophage induction assay

For prophage induction via UV light, overnight cultures were diluted 1:50 (≙ 2%) in 50 ml fresh, preheated (30 °C) mMRS. After the cultures reached an optical density at 600 nm (OD_600_) of 0.3–0.4, 10 ml were transferred into sterile 100 ml Erlenmeyer flasks. Those were put onto a UVT-28 M (Herolab) transilluminator, featuring eight 8 W UV-B light tubes (Herolab; Cat. No. 2984400; spectrum optimum at 312 nm wavelength), and treated with UV light for 4 minutes, the light intensity of the transilluminator was set at “high” (equivalent of 100% light intensity). The cultures were shaken periodically while induction to ensure homogenous UV light exposure.

For prophage induction via mitomycin C, 15 ml preheated mMRS medium was set with overnight cultures to an OD_600_ = 0.05. After bacterial growth to an OD_600_ = 0.1, different amounts of mitomycin C from Streptomyces (Sigma-Aldrich) were added (20 μg/ml; 10 μg/ml; 5 μg/ml; 0.5 μg/ml; 0.2 μg/ml; no mitomycin C).

After Induction (UV light or mitomycin C) samples were transferred to a 48 well microplate (1 ml sample volume per well) and OD_600_ was measured with a FLUOstar Omega reader (BMG LABTECH GmbH) for 12 hours every 5 min. Temperature was set at 30 °C. Before each cycle samples were mixed through double orbital shaking at 200 rpm for 5 s.

To optimise phage induction, each lytic lysogen was treated analogously to the induction procedure using mitomycin C, apart from utilising 5 μg/ml, 4 μg/ml, 3 μg/ml, 2 μg/ml, 1 μg/ml and 0 μg/ml mitomycin C for induction.

### Phage purification protocol

Phage containing cell lysates were harvested the next day after an UV induction experiment for purification via precipitation. After sterile filtration with Filtropur S 0.45 μm filters (Sarstedt) 1.2 ml of each sample were supplemented with 300 μl of 20% (w/v) PEG 8000 (Sigma-Aldrich) solution containing 2.5 M NaCl (Carl Roth). After 1 h incubation on ice, samples were centrifuged for 10 min at 4 °C and 13,000 xg. Supernatant was discarded and phage pellets were solubilised in 120 μl of SM buffer with gelatine [42] corresponding to 1/10th of former sample volume. Purified phage samples were stored at 4 °C.

### Electron microscopy

After phage purification, samples were subjected to negative staining transmission electron microscopy (TEM) as described previously by [43]. After application of 5 μl of sample to glow-discharged and carbon-coated copper grids, the samples were blotted on filter paper and washed twice with double-distilled water. After this, they were negatively stained with 2% uranyl acetate for 20 seconds, blotted again and left for air-drying.

For TEM, a Zeiss EM912 with an integrated OMEGA-filter (Zeiss) and a 2 k × 2 k CCD camera (TRS) was used. The microscope was operated at 80 kV in the zero-loss mode.

### Cell disruption and DNA extraction

Extraction of bacterial genomic DNA was performed with the “E.Z.N.A.® Bacterial DNA Kit” (Omega Bio-Tek) according to the manufacturer’s information. Therefore, approximately 0.8 ml to 1.5 ml of overnight cultures (depending on the size of the cell pellet) were used for extraction. During the cell disruption steps the samples were treated as twice in size. Elution was performed with reduced elution buffer volume (like normal sized samples) for higher yield and concentration of purified DNA. 220 μl Lysozyme solution (10 mg/ml Lysozyme (Omega Bio-Tek) solubilised in TE-buffer (1 mM EDTA-dihydrate (vwr™), 10 mM TRIS (Gerbu), pH 8.0) was added to the cell pellet after centrifugation and incubated at 37 °C for 1.5 h. Optional steps for difficult-to-lyse bacteria with glass beads were carried out by using the FastPrep®-24 (20 s, 4 m/s, 24/2) from MP.

### Genome sequencing, assembly and annotation

Whole genome sequencing of purified DNA was performed with Illumina HiSeq technology by Eurofins (Germany). Raw data was assembled using Unicycler version 0.4.8 [44] on usegalaxy.eu. For the assembly default parameters were used and the contig length cutoff for the generation of FASTA files was set to 1000. The genomes were annotated using the NCBI Prokaryotic Genome Annotation Pipeline (PGAP) [45–47].

### Extraction and sequencing of viral DNA

Strains were induced via a 4-minute UV light treatment as described before. 10 ml of phage containing lysates were harvested by centrifugation (6000 xg, 5 min, RT), sterile filtered (Filtropur S filters with 0.45 μm pore size from SARSTEDT AG & Co. KG) and stored at 4 °C until precipitation. Phages were precipitated at 4 °C by using 0.5 M NaCl and 10% (w/v) PEG8000 (final concentrations) over night. After centrifugation (16,000 xg, 30 min, 4 °C), the supernatant was discarded and the phage containing pellet was resuspended in 500 μl SM buffer [48].

Of each enriched sample the amount of protein was quantified after Bradford using Coomassie Protein Assay Reagent (Thermo Scientific). The standard curve was constructed using a dilution series of bovine serum albumin (BSA; Thermo Scientific) according to the manufacturer. All samples were prepared as duplicates and measured at 595 nm. The protein amounts within each enriched phage solution are listed in Additional File A3.

For removal of bacterial gDNA and RNA, 1.25 μl DNase I (Quiagen) and 2.5 μl RNase (10 mg/ml; Carl Roth) were added and the samples were incubated at 37 °C for 1 h. Virus capsids were digested by adding 1.25 μl Proteinase K (20 mg/ml; Omega Bio-tek), 25 μl 10% (w/v) sodium dodecyl sulfate (SDS; Serva) stock and 20 μl 0.5 M EDTA (pH 8.0; vwr™) and incubating at 60 °C for 1 h. After letting the samples cool down to RT, DNA of each enriched phage sample was extracted via phenol-chloroform extraction followed by ethanol precipitation (Center for Phage Technology; Protocol slightly modified) [49].

For this, an equal volume of a 1:1 mixture of Roti®-Aqua-Phenol (Carl Roth) and chloroform:isoamyl alcohol 24:1 (Sigma) were added to app. 500 μl enriched phage solution followed by inverting the sample multiple times. After centrifugation (3000 xg, 5 min, RT), the supernatant (SN) was transferred into a new 2 ml microcentrifuge tube. After addition of an equal volume of phenol:(chloroform:isoamyl alcohol), inverting, centrifugation and transferring the SN into a new tube (all as mentioned above), an equal volume of chloroform was added and centrifugation and SN transfer was conducted as above. 1/10th volume of 3 M sodium acetate (Carl Roth) and 2.5 volumes ice cold ethanol (96%, vwr™) were added, followed by mixing and incubation at − 20 °C overnight.

After centrifugation (16,000 xg, 20 min, RT), the SN was removed and 1 ml 70% ethanol (made of 96% stock; vwr) was added, followed by centrifugation (16,000 xg, 2 min, RT). The ethanol wash step was repeated once. The ethanol was then removed and the tubes were left open to allow evaporation of residual ethanol. The DNA pellet was then dissolved in 30 μl Elution Buffer from the E.Z.N.A.® Bacterial DNA Kit (Omega Bio-Tek). DNA quantification was performed with a NanoDrop® ND-1000 (Peqlab) spectrophotometer.

The viral genomes were then sequenced (Eurofins INVIEW Virus Sequencing for dsDNA viruses) and assembled as described for the bacterial genomes. For the viral genomes a 100 bp contig cut-off was used.

### Phylogenetic tree analysis

Alignments were created with CLC Main Workbench version 8.1.4 (Qiagen). For the alignments the default settings were used. Phylogenetic trees were created with MEGA version 10.2.6 [50] by using the neighbor joining method (distance measure: Maximum Composite Likelihood method). Bootstrap analysis was performed using 1000 replicates. Notably, the phylogenetic tree of the gene coding for the tape measure protein (TMP) is based on only 996 replicates, as 4 of 1000 replicates failed during the bootstrapping process. The nucleotide sequences of the (pro)phage integrase, terminase (large subunit) and tape measure protein (TMP; first in 5′ direction, if multiple TMP genes were present) were aligned separately and phylogenetic trees were created for each alignment. To extract the marker gene sequences used as outgroups (originating from phages infecting other lactobacilli), the “extract annotation” feature of CLC Main Workbench was used with the search terms “integrase”, “terminase large subunit” and “tape measure” on the phage sequences listed in the chapter “Availability of data and materials”. The *sakei* phage marker genes were extracted manually.

### PCR-mediated amplification of phage DNA

All PCR-mediated amplifications within this study were carried out using a Taq DNA Core Kit 10 (MP Biomedicals™) in combination with a Mastercycler® gradient (Eppendorf).

One 25 μl reaction consisted of 2.5 μl 10 x reaction buffer with MgCl_2_, 0.5 μl dNTPs mix (10 mM each), 1 μl primermix (10 μM each; Eurofins Genomics), 0.15 μl *Taq* polymerase and 19.85 μl water. As DNA substrate, one μl of washed twice, non-induced *L. sakei* TMW 1.46 cryo stock was used, if not indicated otherwise. The washing was conducted by adding 15 μl of a cryo stock to 135 μl TE-buffer (pH 8.0), followed by centrifugation at 16,000 xg, for 2 min, at ambient temperature. After the second wash step, the cell pellet was resuspended in 15 μl TE-buffer (pH 8.0).

PCR-mediated amplification was performed using the following PCR protocol: (93 °C / 4 min) 1x, (93 °C / 45 s, 53 °C / 1 min, 72 °C / t_E_) 35 x, (72 °C / 10 min) 1x. The Elongation time t_E_ was set to 3 min for the prophage circularization PCR, and to 2 min for viral DNA detection.

To check if circularization of TMW 1.46 P1 occurred, the primers 1.46_P1_LYS_FWD [5′-GCTACAATTCAACCGCTTGGG-3′] and 1.46_P1_INT_REV [5′-CAAAGCATTATCCGCAGAC-3′] were used (expected amplificate length: 0.9 kb). Circularization of TMW 1.46 P2 was verified by amplification with the primers 1.46_P2_INT_FWD [5′-CTGGTGTATCCGGTGAGTCG-3′] and 1.46_P2_LYS_REV [5′-CCGCACATACACAGTCCGATAC-3′] were used (expected amplificate length: 2.3 kb). Successful DNA amplification was checked by sequencing of the amplified DNA products via Illumina (Eurofins Genomics) after purification of the PCR reactions using the E.Z.N.A.® Cycle Pure Kit (Omega Bio-Tek) according to manufacturer’s instructions.

For the viral DNA detection via PCR, 1 μl of *L. sakei* TMW 1.46 lysate was used, which was sterile filtered using Filtropur S filters with 0.45 μm pore size (SARSTEDT AG & Co. KG) 24 h after UV light induction and was digested with DNase I. For DNase I digestion 16 μl lysate, 2 μl of solubilised DNase I (stock solution: 1500 Kunitz units in 550 μl RNase-free water) from the RNase-Free DNase Set (QIAGEN) and 2 μl of 10x DNase I Reaction Buffer (Invitrogen) were incubated for 10 minutes at 37 °C, followed by DNase I inactivation at 75 °C for 10 minutes.

The primers 1.46_P1_INT_FWD [5′-GCTACAATTCAACCGCTTGGG-3′] and 1.46_P1_INT_REV [5′-CAAAGCATTATCCGCAGAC-3′] were used for the detection of TMW 1.46 P1 DNA in the DNase I digested, post induced lysate of *L. sakei* TMW 1.46 (expected amplificate length: 127 bp). Viral DNA of TMW 1.46 P2 was amplified with the primers 1.46_P2_TER_FWD [5′-GAAATGGGAACAGATGGGGC-3′] and 1.46_P2_TER_REV [5′-GGCAAATTGATGGCTTAAATGG-3′] (expected amplificate length: 125 bp). Complete digestion of *L. sakei* TMW 1.46 genomic DNA in the lysates was indicated by a negative result using the primers 616 V [5′-AGAGTTTGATYMTGGCTCAG-3′] and 609R [5′-ACTACYVGGGTATCTAAKCC-3′] for partial amplification of the 16 s rDNA genes [51] (expected amplificate length: 825 bp).

### Programs

CLC Main Workbench version 8.1.4 (Qiagen) for BLAST, (whole genome) alignments, phylogenetic trees and bootstrap analyses. SnapGene Viewer version 5.2.3: SnapGene software (from Insightful Science; available at www.snapgene.com) was used to colourise prophage genes according to their predicted function. Easyfig 2.2.5 [52] was used for prophage genome comparison.

### Accession numbers of genomic sequences

Accession numbers of already published *Latilactobacillus sakei* genomes in addition to the genomes of phages, used as outgroups in the construction of phylogenetic trees, are listed in the section “Availability of data and materials” below.

### Detection of prophages, further annotation and prophage visualisation.

An initial genome similarity check was performed to exclude identical genomes from the analysis. This was done by determining the average nucleotide identity based on BLAST (ANIb) over the aligned nucleotides (both in percent) using the web tool JSpeciesWS [53]. Pairwise compared genomes with 100.00% ANIb over a minimum of 99.5% of aligned nucleotides were seen as identical.

For the detection, evaluation of completeness (rating prophages as intact, questionable and incomplete) and a first annotation of prophage sequences in analysed *Latilactobacillus sakei* genomes, the free web application “Phage search tool enhanced release” (PHASTER) by [11, 12] was used. Annotations by PHASTER were checked manually by comparing the translated amino acid sequences received by PHASTER against UniProt’s virus data base (uniprot.org; BLASTp; E-Threshold: 10; Matrix: Auto; Filtering: None; Gapped: yes; Hits: 250) and replaced where reasonable. For the prophage sequence visualisation and allocation of gene module colours the program SnapGene Viewer (Insightful Science; snapgene.com) was used. The colour coding in this study was based on the colour coding of previous works with *Levilactobacillus brevis* phages [15]. The phage sequences within Fig. 1 were presorted by utilising the “whole genome alignment” feature of CLC Main Workbench version 8.1.4 (Qiagen), followed by a pairwise comparison between each sequence, to ensure sequences with higher similarity were depicted next to each other. The (pro)phages were named after their host in combination with a capitalised “p” for “(pro)phage” and a number to distinguish different (pro)phages within one host. (Pro)phage names were written in non-italic, even when the host is included (e.g. L. sakei TMW 1.46 P1 or TMW 1.46 P1). This is in accordance with the “International Committee on Taxonomy of Viruses” (ICTV) [54].

The following blast settings were used for the prophage genome comparison with Easyfig 2.2.5 [52]: Min. length: 0; Max. e-value: 0.001; Min identity value: 0; Outline blast hits in black: yes; Filter small blast hits/annotations: yes. Genomes were aligned on the left side.

## Supplementary Information

**Additional file 1.** Additional File A1

**Additional file 2.** Additional File A2

**Additional file 3.** Additional File A3

**Additional file 4 Fig. S1** PCR-mediated DNA amplification, verifying circularization of both prophages (TMW 1.46 P1 and TMW 1.46 P2) within a non-induced cryo stock of *L. sakei* TMW 1.46, visualised by agarose gel electrophoresis. “1” + “7”: GeneRuler 1 kb DNA Ladder (Thermo Scientific). “2”: TMW 1.46 P1 circularization. “3”: Negative control for “2” (water instead of a washed cryo culture). “4”: Empty. “5”: TMW 1.46 P2 circularization. “6”: Negative control for “5” (water instead of a washed cryo culture).

**Additional file 5 Fig. S2** PCR-mediated DNA amplification, verifying the presence of virions originating from both prophages (TMW 1.46 P1 and TMW 1.46 P2) in post UV light induced, sterile filtered and DNase I digested lysates of *L. sakei* TMW 1.46, visualised by agarose gel electrophoresis. “1” + “11”: GeneRuler 1 kb DNA Ladder (Thermo Scientific). “2”: Post UV light induced, DNase I treated lysate amplificated with TMW 1.46 P1 marker. “3”: Washed *L. s.* TMW 1.46 cryo stock amplificated with TMW 1.46 P1 marker. “4”: Negative control for “2” + “3” (water instead of a washed cryo culture). “5”: Post UV light induced, DNase I treated lysate amplificated with TMW 1.46 P2 marker. “6”: Washed *L. s.* TMW 1.46 cryo stock amplificated with TMW 1.46 P2 marker. “7”: Negative control for “5” + “6” (water instead of a washed cryo culture). “8”: Post UV light induced, DNase I treated lysate amplificated with 16 s rDNA marker. “9”: Washed *L. s.* TMW 1.46 cryo stock amplificated with 16 s rDNA marker. “10”: Negative control for “8” + “9” (water instead of a washed.

**Additional file 6 Fig. S3** Neighbor joining tree of the phage terminase (large subunit) gene of as intact predicted prophages in *L. sakei* strains. Bootstrap values are based on a Jukes-Cantor model (1000 replicates). The phage terminase (large subunit) genes of phages infecting other lactobacilli were included as outgroups.

**Additional file 7 Fig. S4** Neighbor joining tree of the phage tape measure protein (TMP) gene of as intact predicted prophages in *L. sakei* strains. Bootstrap values are based on a Jukes-Cantor model (996 replicates). The TMP genes of phages infecting other lactobacilli were included as outgroups.

**Additional file 8 Table S1** Examples of genes/putative tasks of annotated proteins within the different phage gene modules and their respective color-coding used in the genome comparison.

## Data Availability

The following lactobacilli phage genomes (sorted after host species), used for the construction of phylogenetic trees, can be accessed under their respective accession number at the NCBI website: *Levilactobacillus brevis*: 3–521 (MK504444.1), 3-SAC12 (MK504442.1), 521B (MK504443.1), ATCCB (MK504445.1), JNU P2 (MN830254), JNU P4 (MN830255), Lb (MG020111.1), LBR48 (GU967410.1), SA-C12 (KU052488.1), SAC12B (MK504446.1). *Lacticaseibacillus casei*: A2 (NC_004112.1), J-1 (KC171646.1), LJ (MF999224.1), phiAT3 (NC_005893.1), PL-1 (KC171647.1), PLE2 (KU848187.1), PLE3 (KU848186.1). *Lactobacillus delbrueckii*: c5 (EU340421.2), JCL1032 (EU409559.1), Ld17 (KJ564037.1), Ld25A (KJ564036.1), Ld3 (KJ564038.1), Ldl1 (KM514685.1), LL-H (EF455602.1), LL-Ku (AY739900.2), PMBT4 (MG913376.1), phiJB (KF188409.1), phiLdb (KF188410.1). *Limosilactobacillus fermentum*: JNU P1 (MN830252), JNU P5 (MN830253), LF1 (HQ141410.1), LfeInf (KP054477.1), LfeSau (KP027015.1), phiPYB5 (GU323708.1). *Lactobacillus gasseri*: JNU P11 (MN830257), JNU P7 (MN830256), KC5a (DQ320509.1), phi jlb1 (KF767351.1), phiadh (AJ131519.1). *Lactobacillus helveticus*: AQ113 (HE956704.1). *Lactobacillus jensenii*: Lv-1 (EU871039.1). *Ligilactobacillus murinus*: phiEF-1.1 (MF041990.1). *Lacticaseibacillus paracasei*: CL1 (KR905066.1), CL2 (KR905067.1), iLp1308 (KR905070.1), iLp84 (KR905069.1), JNU P10 (MN830259), JNU P9 (MN830258), T25 (AP018361.1). *Lactiplantibacillus pentosus* & *Lactiplantibacillus plantarum*: LpeD (MF787246.1). *Lactiplantibacillus plantarum*: ATCC 8014-B1 (JX486087.1), ATCC 8014-B2 (JX486088.1), Bacchae (MG765277.2), Bassarid (MG765275.2), Bromius (MH809531.1), Dionysus (MH809530.1), Iacchus (MH809529.1), LP65 (NC_006565.1), Lpa804 (MG557979.1), Maenad (MG765274.1), Nyseid (MG765276.1), P1 (KX223815.1), P2 (KY381600.1), phig1e (NC_004305.1), phiJL-1 (NC_006936.1), PM411 (MG788324.1), Sabazios (MH809528.1), Satyr (MG744354.1), Semele (MG765279.2), Sha1 (HQ141411.1), Silenus (MG765278.2). *Limosilactobacillus reuteri*: LR1 (MH837542.1), LR2 (MH837543.1). *Lacticaseibacillus rhamnosus*: BH1 (MH983004.1), Lc-Nu (NC_007501.1), Lrm1 (EU246945.1). *Fructilactobacillus sanfranciscensis*: EV3 (LN885237.1). The following *Latilactobacillus sakei* genomes of strains, which have been used within this study, can be accessed under their respective accession number at the NCBI website: 23 K (NC_007576), C21B (NZ_CP043730), C22G (NZ_CP043729), CBA3614 (NZ_CP046037), CBA3635 (NZ_CP059697), DS4 (NZ_CP025839), DSM 20017^T^ (CP017271), E23B (NZ_CP043731), E28G (NZ_CP043728), FAM18311 (NZ_CP020459), FLEC01 (NZ_LT960777), J112 (NZ_LT907933), J156 (NZ_LT907929), J160x1 (NZ_LT907931), J18 (NZ_LT907930), J54 (NZ_LT960790), J64 (NZ_LT960781), LK-145 (NZ_AP017931), LT-13 (NZ_AP017929), LZ217 (NZ_CP032652), MBEL1397 (NZ_CP048116), MFPB16A1401 (NZ_LT960788), MFPB19 (NZ_LT960784), ob4.1 (NZ_CP075489), Probio65 (NZ_CP020806), TMW 1.2 (JAMOWF000000000), TMW 1.3 (CP016465), TMW 1.23 (JAHIAK000000000), TMW 1.46 (CP015487), TMW 1.114 (CP017566), TMW 1.411 (QOSE01000001.1), TMW 1.417 (CP017568), TMW 1.578 (CP017570), TMW 1.1239 (CP017272), TMW 1.1290 (JAHIAJ000000000), TMW 1.1386 (JAHIAI000000000), TMW 1.1393 (JAHIAH000000000), TMW 1.1396 (CP017273), TMW 1.1397 (JAMOWE000000000), TMW 1.1398 (CP017275), TMW 1.1500 (JAMOWD000000000), WiKim0063 (NZ_CP022709), WiKim0072 (NZ_CP025136), WiKim0073 (NZ_CP025203), WiKim0074 (NZ_CP025206), ZFM220 (NZ_CP032633), ZFM225 (NZ_CP032635), ZFM229 (NZ_CP032640). Genomes of *Latilactobacillus sakei* phages, sequenced within this study, can be accessed under their respective accession number at the NCBI website: TMW 1.46 P1 (OP455939), TMW 1.46 P2 (OP455940), TMW 1.1386 P1 (OP455936), TMW 1.1393 P1 (OP455937), TMW 1.1397 P1 (OP455938).
