# Supplementary material for: Distribution, inducibility, and characterisation of prophages in Latilactobacillus sakei
Source: BMC Microbiol. 2022 Nov 8;22:267. doi: 10.1186/s12866-022-02675-y (PMC9641780; doi:10.1186/s12866-022-02675-y)

Pairwise comparison after whole genome alignment of putatively intact prophage genomes found within *Latilactobacillus sakei* genomes:

Upper comparison: Average Nucleotide Identity (ANI) in %.

Lower comparison: Alignment Percentage (AP) in %.


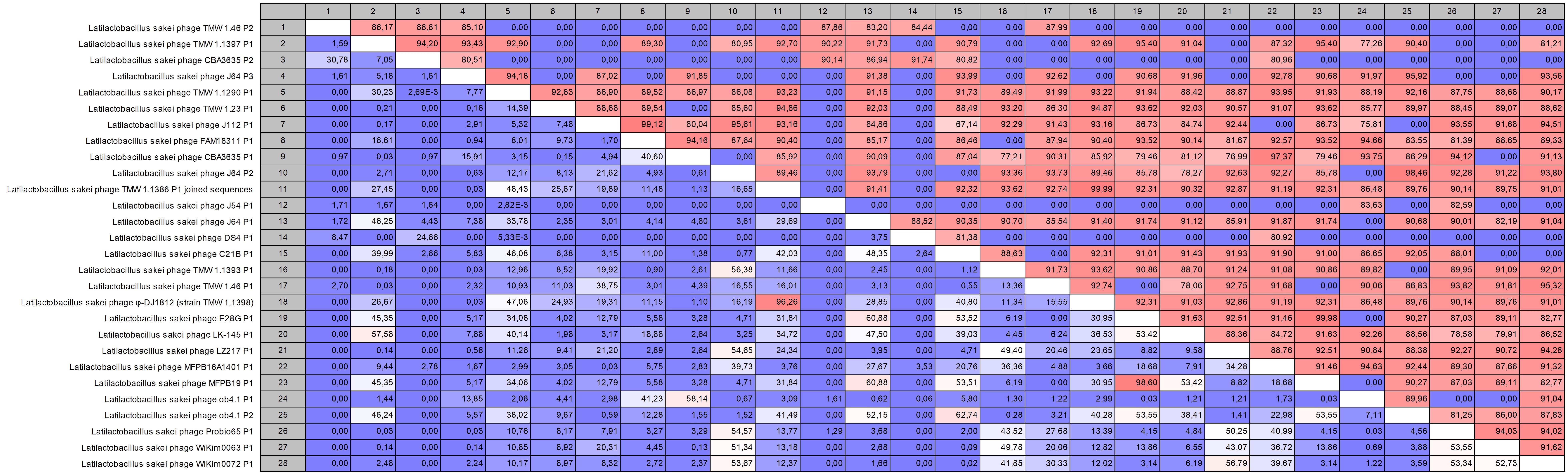


Pairwise comparisons after alignments of *Latilactobacillus* phage integrase genes:

Upper comparison: Percent Identity (The percentage of overlapping alignment positions where the two sequences agree.)

Lower comparison: Distance (The Jukes-Cantor corrected distance between the two sequences)

**Group I (tRNA gene insertion site):**


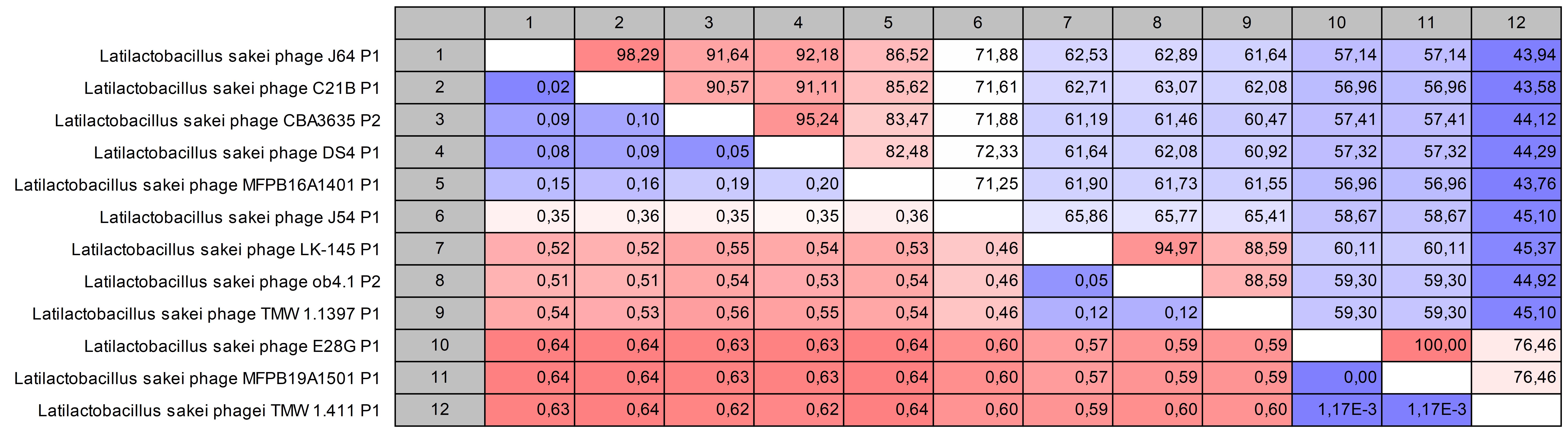


**Group II (tmRNA gene insertion site):**

**
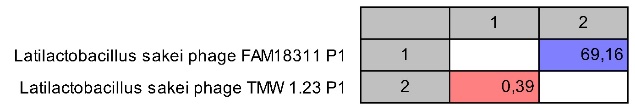
**

**Group III (hypothetical protein gene insertion site):**

**
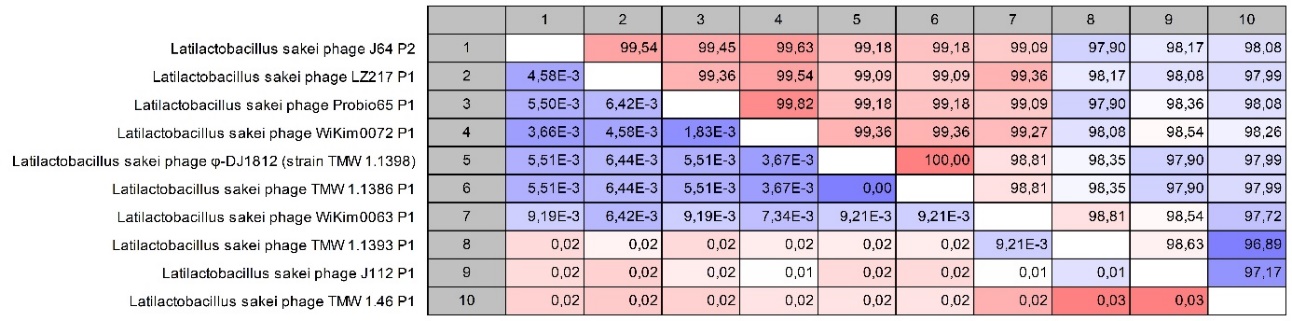
**

**Group IV (SufB gene insertion site):**

**
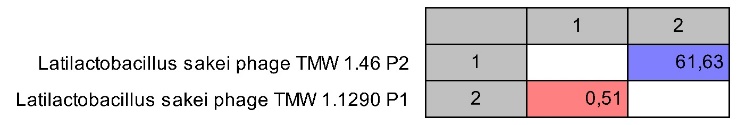
**

**Group V (glutamine-hydrolyzing GMP synthase gene integration site):**

**
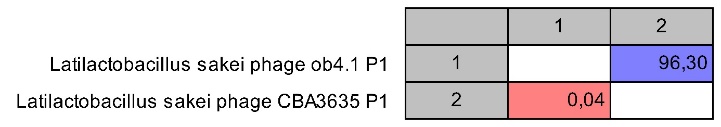
**

**Group VI (glucose-6-phosphate isomerase gene insertion site) (alignment with all other sakei phage integrases):**


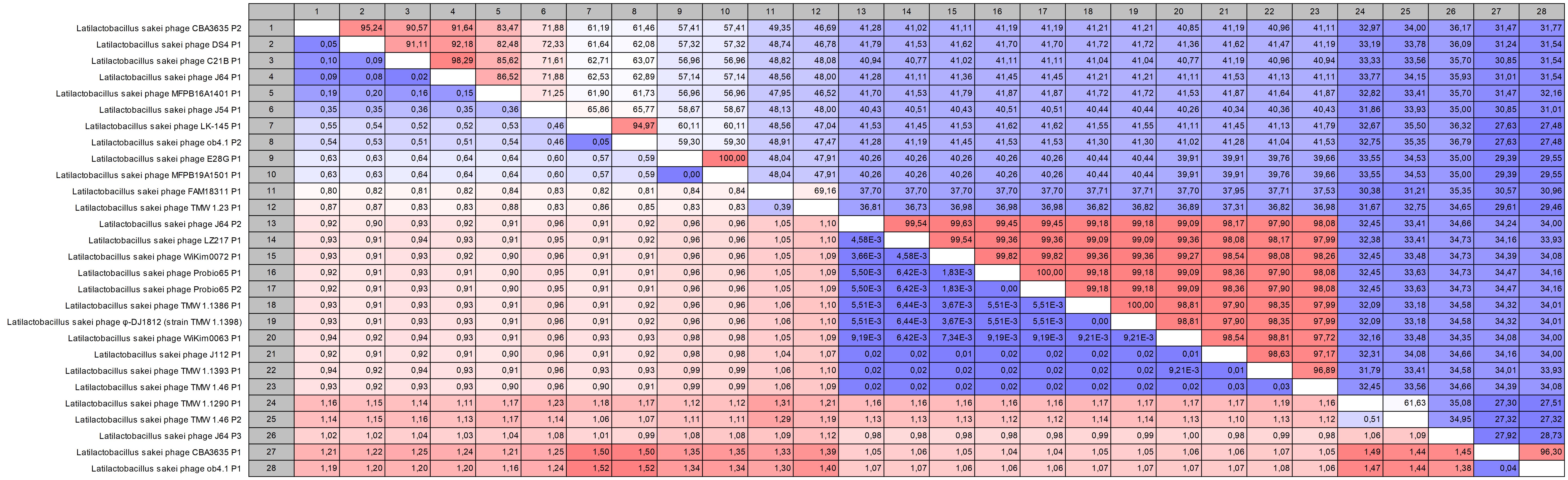

Supplement: Supplementary file 1 — Additional file 1. Additional File A1 [file 12866_2022_2675_MOESM1_ESM.docx]
