# Supplementary material for: Distribution, inducibility, and characterisation of prophages in Latilactobacillus sakei
Source: BMC Microbiol. 2022 Nov 8;22:267. doi: 10.1186/s12866-022-02675-y (PMC9641780; doi:10.1186/s12866-022-02675-y)
Supplement: Supplementary file 2 — Additional file 2. Additional File A2 [file 12866_2022_2675_MOESM2_ESM.docx]

Growth curves of *L. sakei* strains classified as non-inducible after mitomycin C treatment:
