# Supplementary material for: Distribution, inducibility, and characterisation of prophages in Latilactobacillus sakei
Source: BMC Microbiol. 2022 Nov 8;22:267. doi: 10.1186/s12866-022-02675-y (PMC9641780; doi:10.1186/s12866-022-02675-y)
Supplement: Supplementary file 3 — Additional file 3. Additional File A3 [file 12866_2022_2675_MOESM3_ESM.docx]

Virion enrichment, viral DNA extraction, and L. sakei phage TMW 1.1393 P1 DNA digestion (EcoRI)

Spectrophotometric protein quantification (after Bradford), and nucleic acid quantification of enriched phage solution.

**Figure 1** Standard curve constructed using different bovine serum albumin (BSA) concentrations

**Table 1** Spectrophotometric measurements of different bovine serum albumin (BSA) concentrations measured at 595 nm for the construction of the standard curve

|  |  | OD_595_ | |  |  |
| --- | --- | --- | --- | --- | --- |
| Vial | final conc. BSA [µg/ml] | value 1 | value 2 | MEAN | BLANK corrected (-I_MEAN_) |
| A | 2000 | 1.940 | 1.923 | 1.932 | 1.412 |
| B | 1500 | 1.795 | 1.743 | 1.769 | 1.249 |
| C | 1000 | 1.567 | 1.537 | 1.552 | 1.032 |
| D | 750 | 1.346 | 1.322 | 1.334 | 0.814 |
| E | 500 | 1.131 | 1.123 | 1.127 | 0.607 |
| F | 250 | 0.860 | 0.887 | 0.874 | 0.354 |
| G | 125 | 0.705 | 0.715 | 0.710 | 0.190 |
| H | 25 | 0.550 | 0.552 | 0.551 | 0.031 |
| I =BLANK | 0 | 0.517 | 0.523 | 0.520 |  |

**Table 2** Protein concentrations of enriched phage solutions obtained after PEG8000/NaCl precipitation calculated by using the formula of Figure 1.

|  | OD_595_ | |  |  |  |
| --- | --- | --- | --- | --- | --- |
| Phage solutions (*L. sakei* strain) | value 1 | value 2 | OD_595_  MEAN | BLANK corrected  OD_595_ | final protein conc. calculated [µg/ml] |
| TMW 1.23 | 0.624 | 0.612 | 0.618 | 0.098 | 81.6 |
| TMW 1.46 | 0.664 | 0.660 | 0.662 | 0.142 | 96.4 |
| TMW 1.1290 | 1.162 | 1.179 | 1.171 | 0.651 | 504.9 |
| TMW 1.1386 | 0.842 | 0.856 | 0.849 | 0.329 | 195.9 |
| TMW 1.1393 | 0.795 | 0.797 | 0.796 | 0.276 | 161.7 |
| TMW 1.1397 | 0.635 | 0.640 | 0.638 | 0.118 | 87.8 |
| TMW 1.1398 | 0.936 | 0.961 | 0.949 | 0.429 | 272.9 |

**Table 3** Amount and purity of extracted DNA measured with the NanoDrop® ND-1000 (Peqlab) spectrophotometer after phenol chloroform extraction of enriched phage solutions.

| *L. sakei* strain | c(DNA) [ng/µl] | A_260_/A_280_ | A_260_/A_230_ |
| --- | --- | --- | --- |
| TMW 1.23 | 42.6 | 1.82 | 1.66 |
| TMW 1.46 | 10.2 | 1.64 | 1.40 |
| TMW 1.1290 | 1385.5 | 1.99 | 2.44 |
| TMW 1.1386 | 260.2 | 1.95 | 2.13 |
| TMW 1.1393 | 150.7 | 1.89 | 2.11 |
| TMW 1.1397 | 93.2 | 1.89 | 2.12 |
| TMW 1.1398 | 396.0 | 1.97 | 2.41 |

EcoRI digestion of TMW 1.1393 P1 DNA after extraction from an enriched phage solution as proof of genome circularity.


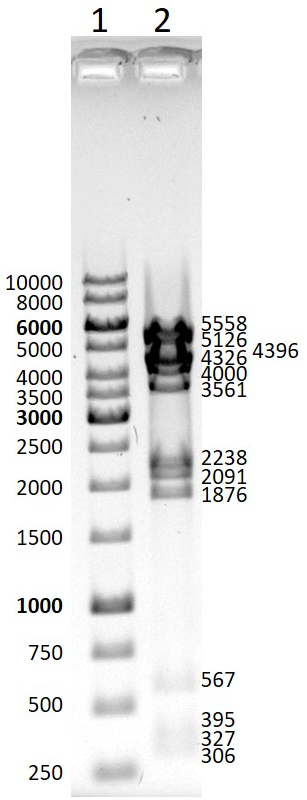


**Figure 2** Digestion of viral TMW 1.1393 P1 DNA via EcoRI. 1: GeneRuler 1 kb DNA Ladder (Thermo Scientific). 2: 800 ng viral TMW 1.1393 P1 DNA + EcoRI (fast digest; Thermo Scientific). All expected fragments (fragment lengths including overhang: 306, 327, 395, 567, 1876, 2091, 2238, 3561, 4000, 4326, 4396, 5126, 5558) were present after digestion via EcoRI, indicating circularity of the phage genome inside the virions.
