# Supplementary material for: Distribution, inducibility, and characterisation of prophages in Latilactobacillus sakei
Source: BMC Microbiol. 2022 Nov 8;22:267. doi: 10.1186/s12866-022-02675-y (PMC9641780; doi:10.1186/s12866-022-02675-y)
Supplement: Supplementary file 4 — Additional file 4 Fig. S1 PCR-mediated DNA amplification, verifying circularization of both prophages (TMW 1.46 P1 and TMW 1.46 P2) within a non-induced cryo stock of L. sakei TMW 1.46, visualised by agarose gel electrophoresis. “1” + “7”: GeneRuler 1 kb DNA Ladder (Thermo Scientific). “2”: TMW 1.46 P1 circularization. “3”: Negative control for “2” (water instead of a washed cryo culture). “4”: Empty. “5”: TMW 1.46 P2 circularization. “6”: Negative control for “5” (water instead of a washed cryo culture). [file 12866_2022_2675_MOESM4_ESM.docx]

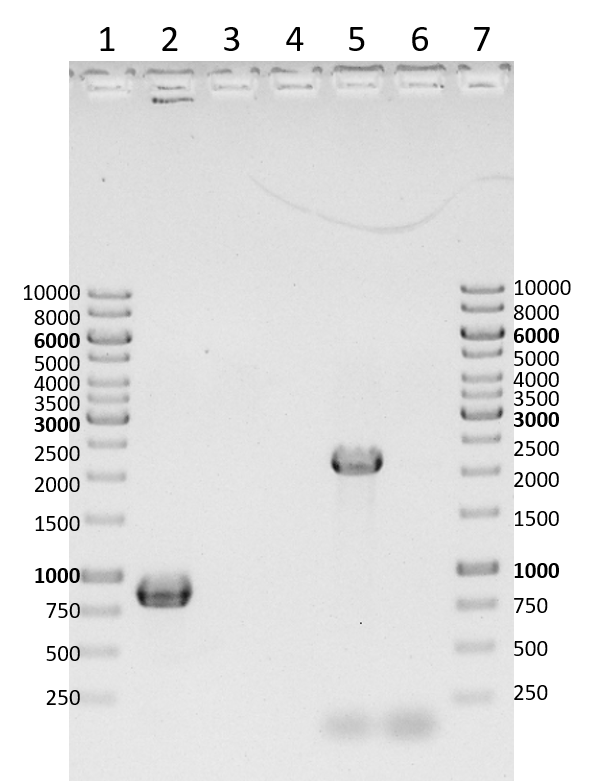


**Figure S1** PCR-mediated DNA amplification, verifying circularization of both prophages (TMW 1.46 P1 and TMW 1.46 P2) within a non-induced cryo stock of *L. sakei* TMW 1.46, visualised by agarose gel electrophoresis. “1” + “7”: GeneRuler 1 kb DNA Ladder (Thermo Scientific). “2”: TMW 1.46 P1 circularization. “3”: Negative control for “2” (water instead of a washed cryo culture). “4”: Empty. “5”: TMW 1.46 P2 circularization. “6”: Negative control for “5” (water instead of a washed cryo culture).
