# Supplementary material for: Distribution, inducibility, and characterisation of prophages in Latilactobacillus sakei
Source: BMC Microbiol. 2022 Nov 8;22:267. doi: 10.1186/s12866-022-02675-y (PMC9641780; doi:10.1186/s12866-022-02675-y)
Supplement: Supplementary file 5 — Additional file 5 Fig. S2 PCR-mediated DNA amplification, verifying the presence of virions originating from both prophages (TMW 1.46 P1 and TMW 1.46 P2) in post UV light induced, sterile filtered and DNase I digested lysates of L. sakei TMW 1.46, visualised by agarose gel electrophoresis. “1” + “11”: GeneRuler 1 kb DNA Ladder (Thermo Scientific). “2”: Post UV light induced, DNase I treated lysate amplificated with TMW 1.46 P1 marker. “3”: Washed L. s. TMW 1.46 cryo stock amplificated with TMW 1.46 P1 marker. “4”: Negative control for “2” + “3” (water instead of a washed cryo culture). “5”: Post UV light induced, DNase I treated lysate amplificated with TMW 1.46 P2 marker. “6”: Washed L. s. TMW 1.46 cryo stock amplificated with TMW 1.46 P2 marker. “7”: Negative control for “5” + “6” (water instead of a washed cryo culture). “8”: Post UV light induced, DNase I treated lysate amplificated with 16 s rDNA marker. “9”: Washed L. s. TMW 1.46 cryo stock amplificated with 16 s rDNA marker. “10”: Negative control for “8” + “9” (water instead of a washed. [file 12866_2022_2675_MOESM5_ESM.docx]

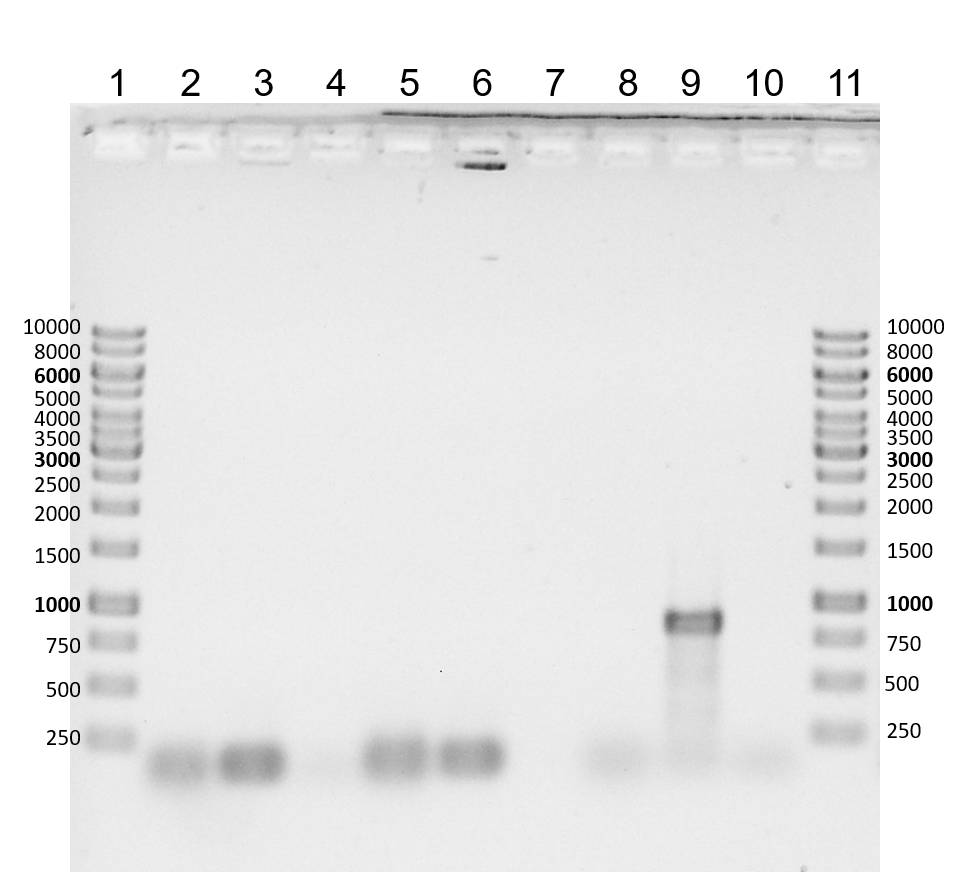


**Figure S2** PCR-mediated DNA amplification, verifying the presence of virions originating from both prophages (TMW 1.46 P1 and TMW 1.46 P2) in post UV light induced, sterile filtered and DNase I digested lysates of *L. sakei* TMW 1.46, visualised by agarose gel electrophoresis. “1” + “11”: GeneRuler 1 kb DNA Ladder (Thermo Scientific). “2”: Post UV light induced, DNase I treated lysate amplificated with TMW 1.46 P1 marker. “3”: Washed L. s. TMW 1.46 cryo stock amplificated with TMW 1.46 P1 marker. “4”: Negative control for “2” + “3” (water instead of a washed cryo culture). “5”: Post UV light induced, DNase I treated lysate amplificated with TMW 1.46 P2 marker. “6”: Washed L. s. TMW 1.46 cryo stock amplificated with TMW 1.46 P2 marker. “7”: Negative control for “5” + “6” (water instead of a washed cryo culture). “8”: Post UV light induced, DNase I treated lysate amplificated with 16s rDNA marker. “9”: Washed L. s. TMW 1.46 cryo stock amplificated with 16s rDNA marker. “10”: Negative control for “8” + “9” (water instead of a washed cryo culture).
