# Supplementary material for: Distribution, inducibility, and characterisation of prophages in Latilactobacillus sakei
Source: BMC Microbiol. 2022 Nov 8;22:267. doi: 10.1186/s12866-022-02675-y (PMC9641780; doi:10.1186/s12866-022-02675-y)
Supplement: Supplementary file 6 — Additional file 6 Fig. S3 Neighbor joining tree of the phage terminase (large subunit) gene of as intact predicted prophages in L. sakei strains. Bootstrap values are based on a Jukes-Cantor model (1000 replicates). The phage terminase (large subunit) genes of phages infecting other lactobacilli were included as outgroups. [file 12866_2022_2675_MOESM6_ESM.docx]

Phage terminase (large subunit) neighbor joining tree

**Figure S3** Neighbor joining tree of the phage terminase (large subunit) gene of as intact predicted prophages in *L. sakei* strains. Bootstrap values are based on a Jukes-Cantor model (1000 replicates). The phage terminase (large subunit) genes of phages infecting other lactobacilli were included as outgroups.
