# Supplementary material for: Distribution, inducibility, and characterisation of prophages in Latilactobacillus sakei
Source: BMC Microbiol. 2022 Nov 8;22:267. doi: 10.1186/s12866-022-02675-y (PMC9641780; doi:10.1186/s12866-022-02675-y)
Supplement: Supplementary file 7 — Additional file 7 Fig. S4 Neighbor joining tree of the phage tape measure protein (TMP) gene of as intact predicted prophages in L. sakei strains. Bootstrap values are based on a Jukes-Cantor model (996 replicates). The TMP genes of phages infecting other lactobacilli were included as outgroups. [file 12866_2022_2675_MOESM7_ESM.docx]

Phage tape measure protein (TMP) neighbor joining tree

**Figure S4** Neighbor joining tree of the phage tape measure protein (TMP) gene of as intact predicted prophages in *L. sakei* strains. Bootstrap values are based on a Jukes-Cantor model (996 replicates). The TMP genes of phages infecting other lactobacilli were included as outgroups.
