# Supplementary material for: Distribution, inducibility, and characterisation of prophages in Latilactobacillus sakei
Source: BMC Microbiol. 2022 Nov 8;22:267. doi: 10.1186/s12866-022-02675-y (PMC9641780; doi:10.1186/s12866-022-02675-y)
Supplement: Supplementary file 8 — Additional file 8 Table S1 Examples of genes/putative tasks of annotated proteins within the different phage gene modules and their respective color-coding used in the genome comparison. [file 12866_2022_2675_MOESM8_ESM.docx]

**Table S1** Examples of genes/putative tasks of annotated proteins within the different phage gene modules and their respective color-coding used in the genome comparison.

| Gene module | Color | Examples for genes or putative tasks of annotated proteins |
| --- | --- | --- |
| Lysogeny | Ochre | (Anti-)repressor, (lysis-)activator, integrase, superinfection immunity protein/abi family protein (abortive infection bacteriophage resistance protein), RecA-like recombination and repair protein, putative host-nuclease inhibitor protein |
| Replication | Green | Cro/CI family transcriptional regulator, transcriptional activator, DNA binding, HTH domain-containing protein, DNA repair, (exo‑/endo-) nuclease, DNA replication, gyrase, transcription, primase, polymerase, helicase, ligase, methylase, methyl transferase, peptidase M78 domain-containing protein, resolvase, recombinase/recombination protein, replication initiation, TMhelix containing protein, zinc finger protein |
| Packaging | Lilac | Terminase (small/large subunit) |
| Head | Light blue | Portal protein, head-tail connector/joining, major/minor capsid proteins, caseinolytic proteins, LysM, prohead protease, scaffold protein, DNA packaging protein |
| Tail | Dark blue | Baseplate proteins, „distal“ fiber protein, sheath protein, tail assembly chaperone, tail endopeptidase, tail protein, tail muraminidase, tape measure protein (TMP) |
| Receptor binding | Yellow | Receptor protein |
| Lysis | Red | Amidase, holin, lysin, lysozyme, |
| Hypothetical protein | Grey | - |
| Task unknown | White | N-acetyltransferase domain-containing protein, phage like protein, YopX domain-containing protein |
| Transposase | Light yellow | Transposase |
